# Supplementary material for: HIVprotI: an integrated web based platform for prediction and design of HIV proteins inhibitors
Source: J Cheminform. 2018 Mar 9;10:12. doi: 10.1186/s13321-018-0266-y (PMC5845081; doi:10.1186/s13321-018-0266-y)
Supplement: Supplementary file 2 — Additional file 2. Source code of HIVProtI web server. [file 13321_2018_266_MOESM2_ESM.zip › HIVprotI_Source-code/hivprotI/weka-3-6-6/doc/overview-tree.html]

Class Hierarchy


---


|  |  |  |
| --- | --- | --- |
| **Overview** \| Package \| Class \| **Tree** \| **Deprecated** \| **Index** \| **Help** \| **Weka's home** | |  |
| PREV   NEXT | **FRAMES**    **NO FRAMES**     **All Classes** |  |


---


## Hierarchy For All Packages

**Package Hierarchies:**: weka.associations, weka.associations.gsp, weka.associations.tertius, weka.attributeSelection, weka.classifiers, weka.classifiers.bayes, weka.classifiers.bayes.blr, weka.classifiers.bayes.net, weka.classifiers.bayes.net.estimate, weka.classifiers.bayes.net.search, weka.classifiers.bayes.net.search.ci, weka.classifiers.bayes.net.search.fixed, weka.classifiers.bayes.net.search.global, weka.classifiers.bayes.net.search.local, weka.classifiers.evaluation, weka.classifiers.functions, weka.classifiers.functions.neural, weka.classifiers.functions.pace, weka.classifiers.functions.supportVector, weka.classifiers.lazy, weka.classifiers.lazy.kstar, weka.classifiers.meta, weka.classifiers.meta.nestedDichotomies, weka.classifiers.mi, weka.classifiers.mi.supportVector, weka.classifiers.misc, weka.classifiers.pmml.consumer, weka.classifiers.rules, weka.classifiers.rules.part, weka.classifiers.trees, weka.classifiers.trees.adtree, weka.classifiers.trees.ft, weka.classifiers.trees.j48, weka.classifiers.trees.lmt, weka.classifiers.trees.m5, weka.classifiers.xml, weka.clusterers, weka.clusterers.forOPTICSAndDBScan.Databases, weka.clusterers.forOPTICSAndDBScan.DataObjects, weka.clusterers.forOPTICSAndDBScan.OPTICS\_GUI, weka.clusterers.forOPTICSAndDBScan.Utils, weka.core, weka.core.converters, weka.core.logging, weka.core.mathematicalexpression, weka.core.matrix, weka.core.neighboursearch, weka.core.neighboursearch.balltrees, weka.core.neighboursearch.covertrees, weka.core.neighboursearch.kdtrees, weka.core.pmml, weka.core.stemmers, weka.core.tokenizers, weka.core.xml, weka.datagenerators, weka.datagenerators.classifiers.classification, weka.datagenerators.classifiers.regression, weka.datagenerators.clusterers, weka.estimators, weka.experiment, weka.experiment.xml, weka.filters, weka.filters.supervised.attribute, weka.filters.supervised.instance, weka.filters.unsupervised.attribute, weka.filters.unsupervised.instance, weka.filters.unsupervised.instance.subsetbyexpression, weka.gui, weka.gui.arffviewer, weka.gui.beans, weka.gui.beans.xml, weka.gui.boundaryvisualizer, weka.gui.experiment, weka.gui.explorer, weka.gui.graphvisualizer, weka.gui.hierarchyvisualizer, weka.gui.sql, weka.gui.sql.event, weka.gui.streams, weka.gui.treevisualizer, weka.gui.visualize, weka.gui.visualize.plugins

---

## Class Hierarchy

- java.lang.Object
  - weka.associations.**AbstractAssociator** (implements weka.associations.Associator, weka.core.CapabilitiesHandler, java.lang.Cloneable, weka.core.RevisionHandler, java.io.Serializable)
    - weka.associations.**Apriori** (implements weka.associations.CARuleMiner, weka.core.OptionHandler, weka.core.TechnicalInformationHandler)- weka.associations.**FPGrowth** (implements weka.core.OptionHandler, weka.core.TechnicalInformationHandler)- weka.associations.**GeneralizedSequentialPatterns** (implements weka.core.OptionHandler, weka.core.TechnicalInformationHandler)- weka.associations.**PredictiveApriori** (implements weka.associations.CARuleMiner, weka.core.OptionHandler, weka.core.TechnicalInformationHandler)- weka.associations.**SingleAssociatorEnhancer** (implements weka.core.OptionHandler)
              - weka.associations.**FilteredAssociator**
              - weka.associations.**Tertius** (implements weka.core.OptionHandler, java.lang.Runnable, weka.core.TechnicalInformationHandler)
    - weka.clusterers.**AbstractClusterer** (implements weka.core.CapabilitiesHandler, java.lang.Cloneable, weka.clusterers.Clusterer, weka.core.RevisionHandler, java.io.Serializable)
      - weka.clusterers.**AbstractDensityBasedClusterer** (implements weka.clusterers.DensityBasedClusterer)
        - weka.clusterers.**MakeDensityBasedClusterer** (implements weka.clusterers.NumberOfClustersRequestable, weka.core.OptionHandler, weka.core.WeightedInstancesHandler)- weka.clusterers.**RandomizableDensityBasedClusterer** (implements weka.core.OptionHandler, weka.core.Randomizable)
            - weka.clusterers.**EM** (implements weka.clusterers.NumberOfClustersRequestable, weka.core.WeightedInstancesHandler)
        - weka.clusterers.**CLOPE** (implements weka.core.OptionHandler, weka.core.TechnicalInformationHandler)- weka.clusterers.**DBScan** (implements weka.core.OptionHandler, weka.core.TechnicalInformationHandler)- weka.clusterers.**HierarchicalClusterer** (implements weka.core.CapabilitiesHandler, weka.core.Drawable, weka.core.OptionHandler)- weka.clusterers.**OPTICS** (implements weka.core.OptionHandler, weka.core.TechnicalInformationHandler)- weka.clusterers.**RandomizableClusterer** (implements weka.core.OptionHandler, weka.core.Randomizable)
                  - weka.clusterers.**Cobweb** (implements weka.core.Drawable, weka.core.TechnicalInformationHandler, weka.clusterers.UpdateableClusterer)- weka.clusterers.**FarthestFirst** (implements weka.core.TechnicalInformationHandler)- weka.clusterers.**sIB** (implements weka.core.TechnicalInformationHandler)- weka.clusterers.**SimpleKMeans** (implements weka.clusterers.NumberOfClustersRequestable, weka.core.WeightedInstancesHandler)- weka.clusterers.**XMeans** (implements weka.core.TechnicalInformationHandler)
                  - weka.clusterers.**RandomizableSingleClustererEnhancer** (implements weka.core.OptionHandler, weka.core.Randomizable)- weka.clusterers.**SingleClustererEnhancer** (implements weka.core.OptionHandler)
                      - weka.clusterers.**FilteredClusterer**
      - javax.swing.AbstractListModel (implements javax.swing.ListModel, java.io.Serializable)
        - javax.swing.DefaultListModel
          - weka.gui.**CheckBoxList.CheckBoxListModel**
        - weka.core.converters.**AbstractLoader** (implements weka.core.converters.Loader)
          - weka.core.converters.**AbstractFileLoader** (implements weka.core.EnvironmentHandler, weka.core.converters.FileSourcedConverter)
            - weka.core.converters.**ArffLoader** (implements weka.core.converters.BatchConverter, weka.core.converters.IncrementalConverter, weka.core.converters.URLSourcedLoader)- weka.core.converters.**C45Loader** (implements weka.core.converters.BatchConverter, weka.core.converters.IncrementalConverter)- weka.core.converters.**CSVLoader** (implements weka.core.converters.BatchConverter, weka.core.OptionHandler)- weka.core.converters.**LibSVMLoader** (implements weka.core.converters.BatchConverter, weka.core.converters.URLSourcedLoader)- weka.core.converters.**SerializedInstancesLoader** (implements weka.core.converters.BatchConverter, weka.core.converters.IncrementalConverter)- weka.core.converters.**SVMLightLoader** (implements weka.core.converters.BatchConverter, weka.core.converters.URLSourcedLoader)- weka.core.converters.**XRFFLoader** (implements weka.core.converters.BatchConverter, weka.core.converters.URLSourcedLoader)
            - weka.core.converters.**DatabaseLoader** (implements weka.core.converters.BatchConverter, weka.core.converters.DatabaseConverter, weka.core.converters.IncrementalConverter, weka.core.OptionHandler)- weka.core.converters.**TextDirectoryLoader** (implements weka.core.converters.BatchConverter, weka.core.OptionHandler)
          - weka.core.converters.**AbstractSaver** (implements weka.core.CapabilitiesHandler, weka.core.converters.Saver)
            - weka.core.converters.**AbstractFileSaver** (implements weka.core.EnvironmentHandler, weka.core.converters.FileSourcedConverter, weka.core.OptionHandler)
              - weka.core.converters.**ArffSaver** (implements weka.core.converters.BatchConverter, weka.core.converters.IncrementalConverter)- weka.core.converters.**C45Saver** (implements weka.core.converters.BatchConverter, weka.core.converters.IncrementalConverter, weka.core.OptionHandler)- weka.core.converters.**CSVSaver** (implements weka.core.converters.BatchConverter, weka.core.converters.FileSourcedConverter, weka.core.converters.IncrementalConverter)- weka.core.converters.**LibSVMSaver** (implements weka.core.converters.BatchConverter, weka.core.converters.IncrementalConverter)- weka.core.converters.**SerializedInstancesSaver** (implements weka.core.converters.BatchConverter)- weka.core.converters.**SVMLightSaver** (implements weka.core.converters.BatchConverter, weka.core.converters.IncrementalConverter)- weka.core.converters.**XRFFSaver** (implements weka.core.converters.BatchConverter)
              - weka.core.converters.**DatabaseSaver** (implements weka.core.converters.BatchConverter, weka.core.converters.DatabaseConverter, weka.core.converters.IncrementalConverter, weka.core.OptionHandler)
            - javax.swing.table.AbstractTableModel (implements java.io.Serializable, javax.swing.table.TableModel)
              - weka.clusterers.forOPTICSAndDBScan.OPTICS\_GUI.**ResultVectorTableModel** (implements weka.core.RevisionHandler)- weka.gui.**SortedTableModel** (implements javax.swing.event.TableModelListener)
                  - weka.gui.arffviewer.**ArffSortedTableModel** (implements weka.core.Undoable)
              - weka.classifiers.bayes.net.**ADNode** (implements weka.core.RevisionHandler, java.io.Serializable, weka.core.TechnicalInformationHandler)- weka.core.**AlgVector** (implements java.lang.Cloneable, weka.core.RevisionHandler, java.io.Serializable)- weka.core.converters.**ArffLoader.ArffReader** (implements weka.core.RevisionHandler)- weka.gui.arffviewer.**ArffTableModel** (implements javax.swing.table.TableModel, weka.core.Undoable)- weka.attributeSelection.**ASEvaluation** (implements weka.core.CapabilitiesHandler, weka.core.RevisionHandler, java.io.Serializable)
                        - weka.attributeSelection.**AttributeSetEvaluator**- weka.attributeSelection.**CfsSubsetEval** (implements weka.core.OptionHandler, weka.attributeSelection.SubsetEvaluator, weka.core.TechnicalInformationHandler)- weka.attributeSelection.**ChiSquaredAttributeEval** (implements weka.attributeSelection.AttributeEvaluator, weka.core.OptionHandler)- weka.attributeSelection.**ConsistencySubsetEval** (implements weka.attributeSelection.SubsetEvaluator, weka.core.TechnicalInformationHandler)- weka.attributeSelection.**CostSensitiveASEvaluation** (implements weka.core.OptionHandler, java.io.Serializable)
                                  - weka.attributeSelection.**CostSensitiveAttributeEval** (implements weka.attributeSelection.AttributeEvaluator, weka.core.OptionHandler, java.io.Serializable)- weka.attributeSelection.**CostSensitiveSubsetEval** (implements weka.core.OptionHandler, java.io.Serializable, weka.attributeSelection.SubsetEvaluator)
                                  - weka.attributeSelection.**FilteredAttributeEval** (implements weka.attributeSelection.AttributeEvaluator, weka.core.OptionHandler, java.io.Serializable)- weka.attributeSelection.**FilteredSubsetEval** (implements weka.core.OptionHandler, java.io.Serializable, weka.attributeSelection.SubsetEvaluator)- weka.attributeSelection.**GainRatioAttributeEval** (implements weka.attributeSelection.AttributeEvaluator, weka.core.OptionHandler)- weka.attributeSelection.**HoldOutSubsetEvaluator** (implements weka.attributeSelection.SubsetEvaluator)
                                          - weka.attributeSelection.**ClassifierSubsetEval** (implements weka.attributeSelection.ErrorBasedMeritEvaluator, weka.core.OptionHandler)
                                          - weka.attributeSelection.**InfoGainAttributeEval** (implements weka.attributeSelection.AttributeEvaluator, weka.core.OptionHandler)- weka.attributeSelection.**OneRAttributeEval** (implements weka.attributeSelection.AttributeEvaluator, weka.core.OptionHandler)- weka.attributeSelection.**ReliefFAttributeEval** (implements weka.attributeSelection.AttributeEvaluator, weka.core.OptionHandler, weka.core.TechnicalInformationHandler)- weka.attributeSelection.**SVMAttributeEval** (implements weka.attributeSelection.AttributeEvaluator, weka.core.OptionHandler, weka.core.TechnicalInformationHandler)- weka.attributeSelection.**SymmetricalUncertAttributeEval** (implements weka.attributeSelection.AttributeEvaluator, weka.core.OptionHandler)- weka.attributeSelection.**UnsupervisedAttributeEvaluator** (implements weka.attributeSelection.AttributeEvaluator)
                                                      - weka.attributeSelection.**LatentSemanticAnalysis** (implements weka.attributeSelection.AttributeTransformer, weka.core.OptionHandler)- weka.attributeSelection.**PrincipalComponents** (implements weka.attributeSelection.AttributeTransformer, weka.core.OptionHandler)
                                                      - weka.attributeSelection.**UnsupervisedSubsetEvaluator** (implements weka.attributeSelection.SubsetEvaluator)- weka.attributeSelection.**WrapperSubsetEval** (implements weka.core.OptionHandler, weka.attributeSelection.SubsetEvaluator, weka.core.TechnicalInformationHandler)
                        - weka.attributeSelection.**ASSearch** (implements weka.core.RevisionHandler, java.io.Serializable)
                          - weka.attributeSelection.**BestFirst** (implements weka.core.OptionHandler, weka.attributeSelection.StartSetHandler)- weka.attributeSelection.**ExhaustiveSearch** (implements weka.core.OptionHandler)- weka.attributeSelection.**GeneticSearch** (implements weka.core.OptionHandler, weka.attributeSelection.StartSetHandler, weka.core.TechnicalInformationHandler)- weka.attributeSelection.**GreedyStepwise** (implements weka.core.OptionHandler, weka.attributeSelection.RankedOutputSearch, weka.attributeSelection.StartSetHandler)- weka.attributeSelection.**LinearForwardSelection** (implements weka.core.OptionHandler, weka.attributeSelection.StartSetHandler, weka.core.TechnicalInformationHandler)- weka.attributeSelection.**RaceSearch** (implements weka.core.OptionHandler, weka.attributeSelection.RankedOutputSearch, weka.core.TechnicalInformationHandler)- weka.attributeSelection.**RandomSearch** (implements weka.core.OptionHandler, weka.attributeSelection.StartSetHandler, weka.core.TechnicalInformationHandler)- weka.attributeSelection.**Ranker** (implements weka.core.OptionHandler, weka.attributeSelection.RankedOutputSearch, weka.attributeSelection.StartSetHandler)- weka.attributeSelection.**RankSearch** (implements weka.core.OptionHandler, weka.core.TechnicalInformationHandler)- weka.attributeSelection.**ScatterSearchV1** (implements weka.core.OptionHandler, weka.core.TechnicalInformationHandler)- weka.attributeSelection.**SubsetSizeForwardSelection** (implements weka.core.OptionHandler)
                          - weka.associations.**AssociatorEvaluation** (implements weka.core.RevisionHandler)- weka.core.**Attribute** (implements weka.core.Copyable, weka.core.RevisionHandler, java.io.Serializable)- weka.core.**AttributeExpression** (implements weka.core.RevisionHandler, java.io.Serializable)- weka.core.**AttributeLocator** (implements java.lang.Comparable<T>, weka.core.RevisionHandler, java.io.Serializable)
                                  - weka.core.**RelationalLocator**- weka.core.**StringLocator**
                                  - weka.gui.visualize.**AttributePanelEvent**- weka.attributeSelection.**AttributeSelection** (implements weka.core.RevisionHandler, java.io.Serializable)- weka.core.**AttributeStats** (implements weka.core.RevisionHandler, java.io.Serializable)- weka.experiment.**AveragingResultProducer** (implements weka.core.AdditionalMeasureProducer, weka.core.OptionHandler, weka.experiment.ResultListener, weka.experiment.ResultProducer, weka.core.RevisionHandler)- weka.core.neighboursearch.balltrees.**BallNode** (implements weka.core.RevisionHandler, java.io.Serializable)- weka.core.neighboursearch.balltrees.**BallSplitter** (implements weka.core.OptionHandler, weka.core.RevisionHandler, java.io.Serializable)
                                              - weka.core.neighboursearch.balltrees.**MedianDistanceFromArbitraryPoint** (implements weka.core.TechnicalInformationHandler)- weka.core.neighboursearch.balltrees.**MedianOfWidestDimension** (implements weka.core.OptionHandler, weka.core.TechnicalInformationHandler)- weka.core.neighboursearch.balltrees.**PointsClosestToFurthestChildren** (implements weka.core.TechnicalInformationHandler)
                                              - weka.core.neighboursearch.balltrees.**BallTreeConstructor** (implements weka.core.OptionHandler, weka.core.RevisionHandler, java.io.Serializable)
                                                - weka.core.neighboursearch.balltrees.**BottomUpConstructor** (implements weka.core.TechnicalInformationHandler)- weka.core.neighboursearch.balltrees.**MiddleOutConstructor** (implements weka.core.Randomizable, weka.core.TechnicalInformationHandler)- weka.core.neighboursearch.balltrees.**TopDownConstructor** (implements weka.core.TechnicalInformationHandler)
                                                - weka.classifiers.bayes.net.estimate.**BayesNetEstimator** (implements weka.core.OptionHandler, weka.core.RevisionHandler, java.io.Serializable)
                                                  - weka.classifiers.bayes.net.estimate.**MultiNomialBMAEstimator**- weka.classifiers.bayes.net.estimate.**SimpleEstimator**
                                                      - weka.classifiers.bayes.net.estimate.**BMAEstimator**
                                                  - weka.gui.beans.**BeanConnection** (implements java.io.Serializable)- weka.gui.beans.**BeanInstance** (implements java.io.Serializable)- weka.attributeSelection.**BestFirst.Link2** (implements weka.core.RevisionHandler, java.io.Serializable)- weka.gui.graphvisualizer.**BIFParser** (implements weka.gui.graphvisualizer.GraphConstants)- weka.gui.**BrowserHelper**- weka.classifiers.**BVDecompose** (implements weka.core.OptionHandler, weka.core.RevisionHandler, weka.core.TechnicalInformationHandler)- weka.classifiers.**BVDecomposeSegCVSub** (implements weka.core.OptionHandler, weka.core.RevisionHandler, weka.core.TechnicalInformationHandler)- weka.core.**Capabilities** (implements java.lang.Cloneable, weka.core.RevisionHandler, java.io.Serializable)- weka.core.**Check** (implements weka.core.OptionHandler, weka.core.RevisionHandler)
                                                                    - weka.core.**CheckGOE**- weka.core.**CheckOptionHandler**- weka.core.**CheckScheme**
                                                                          - weka.associations.**CheckAssociator** (implements weka.core.RevisionHandler)- weka.attributeSelection.**CheckAttributeSelection**- weka.classifiers.**CheckClassifier**- weka.clusterers.**CheckClusterer**- weka.classifiers.functions.supportVector.**CheckKernel**
                                                                    - weka.estimators.**CheckEstimator** (implements weka.core.OptionHandler, weka.core.RevisionHandler)- weka.estimators.**CheckEstimator.AttrTypes** (implements weka.core.RevisionHandler)- weka.estimators.**CheckEstimator.EstTypes** (implements weka.core.RevisionHandler)- weka.estimators.**CheckEstimator.PostProcessor** (implements weka.core.RevisionHandler)- weka.core.**CheckScheme.PostProcessor** (implements weka.core.RevisionHandler)- weka.classifiers.**CheckSource** (implements weka.core.OptionHandler, weka.core.RevisionHandler)- weka.filters.**CheckSource** (implements weka.core.OptionHandler, weka.core.RevisionHandler)- weka.core.matrix.**CholeskyDecomposition** (implements weka.core.RevisionHandler, java.io.Serializable)- weka.core.**ClassDiscovery** (implements weka.core.RevisionHandler)- weka.core.**ClassDiscovery.StringCompare** (implements java.util.Comparator<T>, weka.core.RevisionHandler)- weka.classifiers.**Classifier** (implements weka.core.CapabilitiesHandler, java.lang.Cloneable, weka.core.OptionHandler, weka.core.RevisionHandler, java.io.Serializable)
                                                                                          - weka.classifiers.trees.**ADTree** (implements weka.core.AdditionalMeasureProducer, weka.core.Drawable, weka.classifiers.IterativeClassifier, weka.core.OptionHandler, weka.core.TechnicalInformationHandler, weka.core.WeightedInstancesHandler)- weka.classifiers.bayes.**AODE** (implements weka.core.OptionHandler, weka.core.TechnicalInformationHandler, weka.classifiers.UpdateableClassifier, weka.core.WeightedInstancesHandler)- weka.classifiers.bayes.**AODEsr** (implements weka.core.OptionHandler, weka.core.TechnicalInformationHandler, weka.classifiers.UpdateableClassifier, weka.core.WeightedInstancesHandler)- weka.classifiers.bayes.**BayesianLogisticRegression** (implements weka.core.OptionHandler, weka.core.TechnicalInformationHandler)- weka.classifiers.bayes.**BayesNet** (implements weka.core.AdditionalMeasureProducer, weka.core.Drawable, weka.core.OptionHandler, weka.core.WeightedInstancesHandler)
                                                                                                    - weka.classifiers.bayes.net.**BIFReader** (implements weka.core.TechnicalInformationHandler)- weka.classifiers.bayes.net.**EditableBayesNet**
                                                                                                        - weka.classifiers.bayes.net.**BayesNetGenerator**
                                                                                                    - weka.classifiers.mi.**CitationKNN** (implements weka.core.MultiInstanceCapabilitiesHandler, weka.core.OptionHandler, weka.core.TechnicalInformationHandler)- weka.classifiers.meta.**ClassificationViaClustering**- weka.classifiers.bayes.**ComplementNaiveBayes** (implements weka.core.OptionHandler, weka.core.TechnicalInformationHandler, weka.core.WeightedInstancesHandler)- weka.classifiers.rules.**ConjunctiveRule** (implements weka.core.OptionHandler, weka.core.WeightedInstancesHandler)- weka.classifiers.trees.**DecisionStump** (implements weka.classifiers.Sourcable, weka.core.WeightedInstancesHandler)- weka.classifiers.rules.**DecisionTable** (implements weka.core.AdditionalMeasureProducer, weka.core.OptionHandler, weka.core.TechnicalInformationHandler, weka.core.WeightedInstancesHandler)
                                                                                                                - weka.classifiers.rules.**DTNB**
                                                                                                                - weka.classifiers.bayes.**DMNBtext** (implements weka.core.OptionHandler, weka.core.TechnicalInformationHandler, weka.classifiers.UpdateableClassifier, weka.core.WeightedInstancesHandler)- weka.classifiers.trees.**FT** (implements weka.core.AdditionalMeasureProducer, weka.core.Drawable, weka.core.OptionHandler, weka.core.TechnicalInformationHandler)- weka.classifiers.functions.**GaussianProcesses** (implements weka.classifiers.IntervalEstimator, weka.core.OptionHandler, weka.core.TechnicalInformationHandler)- weka.classifiers.bayes.**HNB** (implements weka.core.TechnicalInformationHandler)- weka.classifiers.misc.**HyperPipes**- weka.classifiers.lazy.**IB1** (implements weka.core.TechnicalInformationHandler, weka.classifiers.UpdateableClassifier)- weka.classifiers.lazy.**IBk** (implements weka.core.AdditionalMeasureProducer, weka.core.OptionHandler, weka.core.TechnicalInformationHandler, weka.classifiers.UpdateableClassifier, weka.core.WeightedInstancesHandler)- weka.classifiers.trees.**Id3** (implements weka.classifiers.Sourcable, weka.core.TechnicalInformationHandler)- weka.classifiers.functions.**IsotonicRegression** (implements weka.core.WeightedInstancesHandler)- weka.classifiers.trees.**J48** (implements weka.core.AdditionalMeasureProducer, weka.core.Drawable, weka.core.Matchable, weka.core.OptionHandler, weka.classifiers.Sourcable, weka.core.Summarizable, weka.core.TechnicalInformationHandler, weka.core.WeightedInstancesHandler)- weka.classifiers.trees.**J48graft** (implements weka.core.AdditionalMeasureProducer, weka.core.Drawable, weka.core.Matchable, weka.core.OptionHandler, weka.classifiers.Sourcable, weka.core.Summarizable, weka.core.TechnicalInformationHandler, weka.core.WeightedInstancesHandler)- weka.classifiers.rules.**JRip** (implements weka.core.AdditionalMeasureProducer, weka.core.TechnicalInformationHandler, weka.core.WeightedInstancesHandler)- weka.classifiers.lazy.**KStar** (implements weka.classifiers.lazy.kstar.KStarConstants, weka.core.TechnicalInformationHandler, weka.classifiers.UpdateableClassifier)- weka.classifiers.trees.**LADTree** (implements weka.core.AdditionalMeasureProducer, weka.core.Drawable, weka.core.TechnicalInformationHandler)- weka.classifiers.lazy.**LBR** (implements weka.core.TechnicalInformationHandler)- weka.classifiers.functions.**LeastMedSq** (implements weka.core.OptionHandler, weka.core.TechnicalInformationHandler)- weka.classifiers.functions.**LibLINEAR** (implements weka.core.TechnicalInformationHandler)- weka.classifiers.functions.**LibSVM** (implements weka.core.TechnicalInformationHandler)- weka.classifiers.functions.**LinearRegression** (implements weka.core.OptionHandler, weka.core.WeightedInstancesHandler)- weka.classifiers.trees.**LMT** (implements weka.core.AdditionalMeasureProducer, weka.core.Drawable, weka.core.OptionHandler, weka.core.TechnicalInformationHandler)- weka.classifiers.functions.**Logistic** (implements weka.core.OptionHandler, weka.core.TechnicalInformationHandler, weka.core.WeightedInstancesHandler)- weka.classifiers.trees.lmt.**LogisticBase** (implements weka.core.WeightedInstancesHandler)
                                                                                                                                                            - weka.classifiers.trees.ft.**FTtree**
                                                                                                                                                              - weka.classifiers.trees.ft.**FTInnerNode**- weka.classifiers.trees.ft.**FTLeavesNode**- weka.classifiers.trees.ft.**FTNode**
                                                                                                                                                              - weka.classifiers.trees.lmt.**LMTNode**
                                                                                                                                                            - weka.classifiers.trees.m5.**M5Base** (implements weka.core.AdditionalMeasureProducer, weka.core.TechnicalInformationHandler)
                                                                                                                                                              - weka.classifiers.trees.**M5P** (implements weka.core.Drawable)- weka.classifiers.rules.**M5Rules** (implements weka.core.TechnicalInformationHandler)
                                                                                                                                                              - weka.classifiers.mi.**MDD** (implements weka.core.MultiInstanceCapabilitiesHandler, weka.core.OptionHandler, weka.core.TechnicalInformationHandler)- weka.classifiers.mi.**MIDD** (implements weka.core.MultiInstanceCapabilitiesHandler, weka.core.OptionHandler, weka.core.TechnicalInformationHandler)- weka.classifiers.mi.**MILR** (implements weka.core.MultiInstanceCapabilitiesHandler, weka.core.OptionHandler)- weka.classifiers.mi.**MINND** (implements weka.core.MultiInstanceCapabilitiesHandler, weka.core.OptionHandler, weka.core.TechnicalInformationHandler)- weka.classifiers.mi.**MIOptimalBall** (implements weka.core.MultiInstanceCapabilitiesHandler, weka.core.OptionHandler, weka.core.TechnicalInformationHandler, weka.core.WeightedInstancesHandler)- weka.classifiers.mi.**MISMO** (implements weka.core.MultiInstanceCapabilitiesHandler, weka.core.TechnicalInformationHandler, weka.core.WeightedInstancesHandler)- weka.classifiers.mi.**MISVM** (implements weka.core.MultiInstanceCapabilitiesHandler, weka.core.OptionHandler, weka.core.TechnicalInformationHandler)- weka.classifiers.functions.**MultilayerPerceptron** (implements weka.core.OptionHandler, weka.core.Randomizable, weka.core.WeightedInstancesHandler)- weka.classifiers.**MultipleClassifiersCombiner**
                                                                                                                                                                                - weka.classifiers.**RandomizableMultipleClassifiersCombiner** (implements weka.core.Randomizable)
                                                                                                                                                                                  - weka.classifiers.meta.**MultiScheme**- weka.classifiers.meta.**Stacking** (implements weka.core.TechnicalInformationHandler)
                                                                                                                                                                                      - weka.classifiers.meta.**Grading** (implements weka.core.TechnicalInformationHandler)- weka.classifiers.meta.**StackingC** (implements weka.core.OptionHandler, weka.core.TechnicalInformationHandler)
                                                                                                                                                                                      - weka.classifiers.meta.**Vote** (implements weka.core.TechnicalInformationHandler)
                                                                                                                                                                                - weka.classifiers.bayes.**NaiveBayes** (implements weka.core.OptionHandler, weka.core.TechnicalInformationHandler, weka.core.WeightedInstancesHandler)
                                                                                                                                                                                  - weka.classifiers.bayes.**NaiveBayesUpdateable** (implements weka.classifiers.UpdateableClassifier)
                                                                                                                                                                                  - weka.classifiers.bayes.**NaiveBayesMultinomial** (implements weka.core.TechnicalInformationHandler, weka.core.WeightedInstancesHandler)
                                                                                                                                                                                    - weka.classifiers.bayes.**NaiveBayesMultinomialUpdateable** (implements weka.classifiers.UpdateableClassifier)
                                                                                                                                                                                    - weka.classifiers.bayes.**NaiveBayesSimple** (implements weka.core.TechnicalInformationHandler)- weka.classifiers.trees.**NBTree** (implements weka.core.AdditionalMeasureProducer, weka.core.Drawable, weka.core.Summarizable, weka.core.TechnicalInformationHandler, weka.core.WeightedInstancesHandler)- weka.classifiers.rules.**NNge** (implements weka.core.OptionHandler, weka.core.TechnicalInformationHandler, weka.classifiers.UpdateableClassifier)- weka.classifiers.rules.**OneR** (implements weka.classifiers.Sourcable, weka.core.TechnicalInformationHandler)- weka.classifiers.functions.**PaceRegression** (implements weka.core.OptionHandler, weka.core.TechnicalInformationHandler, weka.core.WeightedInstancesHandler)- weka.classifiers.rules.**PART** (implements weka.core.AdditionalMeasureProducer, weka.core.OptionHandler, weka.core.Summarizable, weka.core.TechnicalInformationHandler, weka.core.WeightedInstancesHandler)- weka.classifiers.functions.**PLSClassifier**- weka.classifiers.pmml.consumer.**PMMLClassifier** (implements weka.core.pmml.PMMLModel, java.io.Serializable)
                                                                                                                                                                                                    - weka.classifiers.pmml.consumer.**GeneralRegression** (implements java.io.Serializable)- weka.classifiers.pmml.consumer.**NeuralNetwork**- weka.classifiers.pmml.consumer.**Regression** (implements java.io.Serializable)
                                                                                                                                                                                                    - weka.classifiers.trees.m5.**PreConstructedLinearModel** (implements java.io.Serializable)- weka.classifiers.rules.**Prism** (implements weka.core.TechnicalInformationHandler)- weka.classifiers.trees.**RandomForest** (implements weka.core.AdditionalMeasureProducer, weka.core.OptionHandler, weka.core.Randomizable, weka.core.TechnicalInformationHandler, weka.core.WeightedInstancesHandler)- weka.classifiers.**RandomizableClassifier** (implements weka.core.Randomizable)
                                                                                                                                                                                                            - weka.classifiers.trees.**BFTree** (implements weka.core.AdditionalMeasureProducer, weka.core.TechnicalInformationHandler)- weka.classifiers.mi.**MIEMDD** (implements weka.core.MultiInstanceCapabilitiesHandler, weka.core.OptionHandler, weka.core.TechnicalInformationHandler)- weka.classifiers.trees.**SimpleCart** (implements weka.core.AdditionalMeasureProducer, weka.core.TechnicalInformationHandler)
                                                                                                                                                                                                            - weka.classifiers.trees.**RandomTree** (implements weka.core.Drawable, weka.core.OptionHandler, weka.core.Randomizable, weka.core.WeightedInstancesHandler)- weka.classifiers.functions.**RBFNetwork** (implements weka.core.OptionHandler)- weka.classifiers.trees.**REPTree** (implements weka.core.AdditionalMeasureProducer, weka.core.Drawable, weka.core.OptionHandler, weka.classifiers.Sourcable, weka.core.WeightedInstancesHandler)- weka.classifiers.rules.**Ridor** (implements weka.core.AdditionalMeasureProducer, weka.core.WeightedInstancesHandler)- weka.classifiers.trees.m5.**RuleNode**- weka.classifiers.misc.**SerializedClassifier**- weka.classifiers.functions.**SimpleLinearRegression** (implements weka.core.WeightedInstancesHandler)- weka.classifiers.functions.**SimpleLogistic** (implements weka.core.AdditionalMeasureProducer, weka.core.OptionHandler, weka.core.TechnicalInformationHandler, weka.core.WeightedInstancesHandler)- weka.classifiers.**SingleClassifierEnhancer**
                                                                                                                                                                                                                              - weka.classifiers.meta.**AttributeSelectedClassifier** (implements weka.core.AdditionalMeasureProducer, weka.core.Drawable, weka.core.OptionHandler, weka.core.WeightedInstancesHandler)- weka.classifiers.meta.**ClassificationViaRegression** (implements weka.core.TechnicalInformationHandler)- weka.classifiers.meta.**FilteredClassifier** (implements weka.core.Drawable)- weka.classifiers.**IteratedSingleClassifierEnhancer**
                                                                                                                                                                                                                                      - weka.classifiers.meta.**AdditiveRegression** (implements weka.core.AdditionalMeasureProducer, weka.core.OptionHandler, weka.core.TechnicalInformationHandler, weka.core.WeightedInstancesHandler)- weka.classifiers.**RandomizableIteratedSingleClassifierEnhancer** (implements weka.core.Randomizable)
                                                                                                                                                                                                                                          - weka.classifiers.meta.**AdaBoostM1** (implements weka.classifiers.Sourcable, weka.core.TechnicalInformationHandler, weka.core.WeightedInstancesHandler)
                                                                                                                                                                                                                                            - weka.classifiers.meta.**MultiBoostAB** (implements weka.core.TechnicalInformationHandler)
                                                                                                                                                                                                                                            - weka.classifiers.meta.**Bagging** (implements weka.core.AdditionalMeasureProducer, weka.core.TechnicalInformationHandler, weka.core.WeightedInstancesHandler)- weka.classifiers.meta.**Decorate** (implements weka.core.TechnicalInformationHandler)- weka.classifiers.meta.**END** (implements weka.core.TechnicalInformationHandler)- weka.classifiers.meta.**LogitBoost** (implements weka.classifiers.Sourcable, weka.core.TechnicalInformationHandler, weka.core.WeightedInstancesHandler)- weka.classifiers.meta.**RandomCommittee** (implements weka.core.WeightedInstancesHandler)- weka.classifiers.meta.**RandomSubSpace** (implements weka.core.TechnicalInformationHandler, weka.core.WeightedInstancesHandler)- weka.classifiers.meta.**RotationForest** (implements weka.core.TechnicalInformationHandler, weka.core.WeightedInstancesHandler)
                                                                                                                                                                                                                                      - weka.classifiers.lazy.**LWL** (implements weka.core.TechnicalInformationHandler, weka.classifiers.UpdateableClassifier, weka.core.WeightedInstancesHandler)- weka.classifiers.mi.**MIBoost** (implements weka.core.MultiInstanceCapabilitiesHandler, weka.core.OptionHandler, weka.core.TechnicalInformationHandler)- weka.classifiers.mi.**MIWrapper** (implements weka.core.MultiInstanceCapabilitiesHandler, weka.core.OptionHandler, weka.core.TechnicalInformationHandler)- weka.classifiers.meta.**OrdinalClassClassifier** (implements weka.core.OptionHandler, weka.core.TechnicalInformationHandler)- weka.classifiers.**RandomizableSingleClassifierEnhancer** (implements weka.core.Randomizable)
                                                                                                                                                                                                                                                - weka.classifiers.meta.nestedDichotomies.**ClassBalancedND** (implements weka.core.TechnicalInformationHandler)- weka.classifiers.meta.**CostSensitiveClassifier** (implements weka.core.Drawable, weka.core.OptionHandler)- weka.classifiers.meta.**CVParameterSelection** (implements weka.core.Drawable, weka.core.Summarizable, weka.core.TechnicalInformationHandler)- weka.classifiers.meta.**Dagging** (implements weka.core.TechnicalInformationHandler)- weka.classifiers.meta.nestedDichotomies.**DataNearBalancedND** (implements weka.core.TechnicalInformationHandler)- weka.classifiers.meta.**GridSearch** (implements weka.core.AdditionalMeasureProducer, weka.core.Summarizable)- weka.classifiers.meta.**MetaCost** (implements weka.core.TechnicalInformationHandler)- weka.classifiers.meta.**MultiClassClassifier** (implements weka.core.OptionHandler)- weka.classifiers.meta.nestedDichotomies.**ND** (implements weka.core.TechnicalInformationHandler)- weka.classifiers.meta.**RacedIncrementalLogitBoost** (implements weka.core.TechnicalInformationHandler, weka.classifiers.UpdateableClassifier)- weka.classifiers.meta.**ThresholdSelector** (implements weka.core.Drawable, weka.core.OptionHandler)
                                                                                                                                                                                                                                                - weka.classifiers.meta.**RegressionByDiscretization**- weka.classifiers.mi.**SimpleMI** (implements weka.core.MultiInstanceCapabilitiesHandler, weka.core.OptionHandler)
                                                                                                                                                                                                                              - weka.classifiers.functions.**SMO** (implements weka.core.TechnicalInformationHandler, weka.core.WeightedInstancesHandler)- weka.classifiers.functions.**SMOreg** (implements weka.core.AdditionalMeasureProducer, weka.core.TechnicalInformationHandler, weka.core.WeightedInstancesHandler)- weka.classifiers.functions.**SPegasos** (implements weka.core.OptionHandler, weka.core.TechnicalInformationHandler, weka.classifiers.UpdateableClassifier)- weka.classifiers.trees.**UserClassifier** (implements weka.core.Drawable, weka.core.TechnicalInformationHandler, weka.gui.treevisualizer.TreeDisplayListener, weka.gui.visualize.VisualizePanelListener)- weka.classifiers.misc.**VFI** (implements weka.core.OptionHandler, weka.core.TechnicalInformationHandler, weka.core.WeightedInstancesHandler)- weka.classifiers.functions.**VotedPerceptron** (implements weka.core.OptionHandler, weka.core.TechnicalInformationHandler)- weka.classifiers.bayes.**WAODE** (implements weka.core.TechnicalInformationHandler)- weka.classifiers.functions.**Winnow** (implements weka.core.TechnicalInformationHandler, weka.classifiers.UpdateableClassifier)- weka.classifiers.rules.**ZeroR** (implements weka.classifiers.Sourcable, weka.core.WeightedInstancesHandler)
                                                                                          - weka.classifiers.rules.part.**ClassifierDecList** (implements weka.core.RevisionHandler, java.io.Serializable)
                                                                                            - weka.classifiers.rules.part.**C45PruneableDecList**- weka.classifiers.rules.part.**PruneableDecList**
                                                                                            - weka.experiment.**ClassifierSplitEvaluator** (implements weka.core.AdditionalMeasureProducer, weka.core.OptionHandler, weka.core.RevisionHandler, weka.experiment.SplitEvaluator)
                                                                                              - weka.experiment.**CostSensitiveClassifierSplitEvaluator**
                                                                                              - weka.classifiers.trees.j48.**ClassifierSplitModel** (implements java.lang.Cloneable, weka.core.RevisionHandler, java.io.Serializable)
                                                                                                - weka.classifiers.trees.j48.**BinC45Split**- weka.classifiers.trees.j48.**C45Split**- weka.classifiers.trees.j48.**GraftSplit** (implements java.lang.Comparable<T>)- weka.classifiers.trees.j48.**NBTreeNoSplit**- weka.classifiers.trees.j48.**NBTreeSplit**- weka.classifiers.trees.j48.**NoSplit**- weka.classifiers.trees.lmt.**ResidualSplit**
                                                                                                - weka.classifiers.trees.j48.**ClassifierTree** (implements weka.core.CapabilitiesHandler, weka.core.Drawable, weka.core.RevisionHandler, java.io.Serializable)
                                                                                                  - weka.classifiers.trees.j48.**C45PruneableClassifierTree**- weka.classifiers.trees.j48.**C45PruneableClassifierTreeG**- weka.classifiers.trees.j48.**NBTreeClassifierTree**- weka.classifiers.trees.j48.**PruneableClassifierTree**
                                                                                                  - weka.core.**ClassloaderUtil** (implements weka.core.RevisionHandler)- weka.datagenerators.**ClusterDefinition** (implements weka.core.OptionHandler, weka.core.RevisionHandler, java.io.Serializable)
                                                                                                      - weka.datagenerators.clusterers.**SubspaceClusterDefinition**
                                                                                                      - weka.clusterers.**ClusterEvaluation** (implements weka.core.RevisionHandler, java.io.Serializable)- weka.gui.treevisualizer.**Colors**- java.awt.Component (implements java.awt.image.ImageObserver, java.awt.MenuContainer, java.io.Serializable)
                                                                                                            - java.awt.Container
                                                                                                              - javax.swing.JComponent (implements java.io.Serializable)
                                                                                                                - javax.swing.AbstractButton (implements java.awt.ItemSelectable, javax.swing.SwingConstants)
                                                                                                                  - javax.swing.JToggleButton (implements javax.accessibility.Accessible)
                                                                                                                    - javax.swing.JCheckBox (implements javax.accessibility.Accessible)
                                                                                                                      - weka.gui.**CheckBoxList.CheckBoxListRenderer** (implements javax.swing.ListCellRenderer)
                                                                                                                  - weka.clusterers.forOPTICSAndDBScan.OPTICS\_GUI.**GraphPanel** (implements weka.core.RevisionHandler)- javax.swing.JFileChooser (implements javax.accessibility.Accessible)
                                                                                                                      - weka.gui.**ConverterFileChooser**
                                                                                                                      - javax.swing.JInternalFrame (implements javax.accessibility.Accessible, javax.swing.RootPaneContainer, javax.swing.WindowConstants)
                                                                                                                        - weka.gui.**Main.ChildFrameMDI**
                                                                                                                        - javax.swing.JLabel (implements javax.accessibility.Accessible, javax.swing.SwingConstants)
                                                                                                                          - javax.swing.DefaultListCellRenderer (implements javax.swing.ListCellRenderer, java.io.Serializable)
                                                                                                                            - weka.gui.experiment.**AlgorithmListPanel.ObjectCellRenderer**
                                                                                                                            - javax.swing.table.DefaultTableCellRenderer (implements java.io.Serializable, javax.swing.table.TableCellRenderer)
                                                                                                                              - weka.gui.arffviewer.**ArffTableCellRenderer**- weka.gui.sql.**ResultSetTableCellRenderer**
                                                                                                                              - weka.gui.sql.**InfoPanelCellRenderer** (implements javax.swing.ListCellRenderer)
                                                                                                                          - javax.swing.JLayeredPane (implements javax.accessibility.Accessible)
                                                                                                                            - javax.swing.JDesktopPane (implements javax.accessibility.Accessible)
                                                                                                                              - weka.gui.**Main.BackgroundDesktopPane**
                                                                                                                            - javax.swing.JList (implements javax.accessibility.Accessible, javax.swing.Scrollable)
                                                                                                                              - weka.gui.**CheckBoxList**
                                                                                                                              - javax.swing.JPanel (implements javax.accessibility.Accessible)
                                                                                                                                - weka.gui.beans.**AbstractDataSink** (implements weka.gui.beans.BeanCommon, weka.gui.beans.DataSink, weka.gui.beans.DataSourceListener, weka.gui.beans.InstanceListener, java.io.Serializable, weka.gui.beans.TestSetListener, weka.gui.beans.ThresholdDataListener, weka.gui.beans.TrainingSetListener, weka.gui.beans.Visible)
                                                                                                                                  - weka.gui.beans.**Saver** (implements weka.core.EnvironmentHandler, weka.gui.beans.WekaWrapper)
                                                                                                                                  - weka.gui.beans.**AbstractDataSource** (implements java.beans.beancontext.BeanContextChild, weka.gui.beans.DataSource, java.io.Serializable, weka.gui.beans.Visible)
                                                                                                                                    - weka.gui.beans.**Loader** (implements weka.gui.beans.BeanCommon, weka.core.EnvironmentHandler, weka.gui.beans.EventConstraints, weka.gui.beans.Startable, weka.gui.beans.StructureProducer, weka.gui.beans.WekaWrapper)
                                                                                                                                    - weka.gui.beans.**AbstractEvaluator** (implements weka.gui.beans.BeanCommon, java.io.Serializable, weka.gui.beans.Visible)
                                                                                                                                      - weka.gui.beans.**ClassifierPerformanceEvaluator** (implements weka.gui.beans.BatchClassifierListener, weka.gui.beans.EventConstraints, java.io.Serializable, weka.gui.beans.UserRequestAcceptor)- weka.gui.beans.**ClustererPerformanceEvaluator** (implements weka.gui.beans.BatchClustererListener, weka.gui.beans.EventConstraints, java.io.Serializable, weka.gui.beans.UserRequestAcceptor)- weka.gui.beans.**IncrementalClassifierEvaluator** (implements weka.gui.beans.EventConstraints, weka.gui.beans.IncrementalClassifierListener)
                                                                                                                                      - weka.gui.beans.**AbstractTestSetProducer** (implements weka.gui.beans.BeanCommon, java.io.Serializable, weka.gui.beans.TestSetProducer, weka.gui.beans.Visible)
                                                                                                                                        - weka.gui.beans.**TestSetMaker** (implements weka.gui.beans.DataSourceListener, weka.gui.beans.EventConstraints, java.io.Serializable)
                                                                                                                                        - weka.gui.beans.**AbstractTrainAndTestSetProducer** (implements weka.gui.beans.BeanCommon, weka.gui.beans.DataSourceListener, java.io.Serializable, weka.gui.beans.TestSetProducer, weka.gui.beans.TrainingSetProducer, weka.gui.beans.Visible)
                                                                                                                                          - weka.gui.beans.**CrossValidationFoldMaker** (implements weka.gui.beans.DataSourceListener, weka.gui.beans.EventConstraints, java.io.Serializable, weka.gui.beans.TestSetListener, weka.gui.beans.TrainingSetListener, weka.gui.beans.UserRequestAcceptor)- weka.gui.beans.**TrainTestSplitMaker** (implements weka.gui.beans.DataSourceListener, weka.gui.beans.EventConstraints, java.io.Serializable, weka.gui.beans.TestSetListener, weka.gui.beans.TrainingSetListener, weka.gui.beans.UserRequestAcceptor)
                                                                                                                                          - weka.gui.beans.**AbstractTrainingSetProducer** (implements weka.gui.beans.BeanCommon, java.io.Serializable, weka.gui.beans.TrainingSetProducer, weka.gui.beans.Visible)
                                                                                                                                            - weka.gui.beans.**TrainingSetMaker** (implements weka.gui.beans.DataSourceListener, weka.gui.beans.EventConstraints, java.io.Serializable)
                                                                                                                                            - weka.gui.experiment.**AlgorithmListPanel** (implements java.awt.event.ActionListener)- weka.gui.arffviewer.**ArffPanel** (implements java.awt.event.ActionListener, javax.swing.event.ChangeListener, java.awt.event.MouseListener, weka.core.Undoable)- weka.gui.arffviewer.**ArffViewerMainPanel** (implements java.awt.event.ActionListener, javax.swing.event.ChangeListener)- weka.gui.explorer.**AssociationsPanel** (implements weka.gui.explorer.Explorer.CapabilitiesFilterChangeListener, weka.gui.explorer.Explorer.ExplorerPanel, weka.gui.explorer.Explorer.LogHandler)- weka.gui.beans.**Associator** (implements weka.gui.beans.BeanCommon, weka.gui.beans.DataSourceListener, weka.gui.beans.EventConstraints, java.io.Serializable, weka.gui.beans.TrainingSetListener, weka.gui.beans.UserRequestAcceptor, weka.gui.beans.Visible, weka.gui.beans.WekaWrapper)- weka.gui.beans.**AssociatorCustomizer** (implements java.beans.Customizer, weka.gui.beans.CustomizerCloseRequester)- weka.gui.**AttributeListPanel**- weka.gui.**AttributeSelectionPanel**- weka.gui.explorer.**AttributeSelectionPanel** (implements weka.gui.explorer.Explorer.CapabilitiesFilterChangeListener, weka.gui.explorer.Explorer.ExplorerPanel, weka.gui.explorer.Explorer.LogHandler)- weka.gui.**AttributeSummaryPanel**- weka.gui.beans.**BeanVisual**- weka.gui.boundaryvisualizer.**BoundaryPanel**
                                                                                                                                                                    - weka.gui.boundaryvisualizer.**BoundaryPanelDistributed**
                                                                                                                                                                    - weka.gui.boundaryvisualizer.**BoundaryVisualizer** (implements weka.core.TechnicalInformationHandler)- weka.gui.beans.**ClassAssigner** (implements weka.gui.beans.BeanCommon, weka.gui.beans.DataSource, weka.gui.beans.DataSourceListener, weka.gui.beans.EventConstraints, weka.gui.beans.InstanceListener, java.io.Serializable, weka.gui.beans.StructureProducer, weka.gui.beans.TestSetListener, weka.gui.beans.TestSetProducer, weka.gui.beans.TrainingSetListener, weka.gui.beans.TrainingSetProducer, weka.gui.beans.Visible)- weka.gui.beans.**ClassAssignerCustomizer** (implements java.beans.Customizer, weka.gui.beans.CustomizerCloseRequester, weka.gui.beans.CustomizerClosingListener, weka.gui.beans.DataFormatListener)- weka.gui.beans.**Classifier** (implements weka.gui.beans.BeanCommon, weka.gui.beans.EventConstraints, weka.gui.beans.InstanceListener, java.io.Serializable, weka.gui.beans.TestSetListener, weka.gui.beans.TrainingSetListener, weka.gui.beans.UserRequestAcceptor, weka.gui.beans.Visible, weka.gui.beans.WekaWrapper)- weka.gui.beans.**ClassifierCustomizer** (implements java.beans.Customizer, weka.gui.beans.CustomizerCloseRequester, weka.gui.beans.CustomizerClosingListener)- weka.gui.explorer.**ClassifierPanel** (implements weka.gui.explorer.Explorer.CapabilitiesFilterChangeListener, weka.gui.explorer.Explorer.ExplorerPanel, weka.gui.explorer.Explorer.LogHandler)- weka.gui.visualize.**ClassPanel**- weka.gui.beans.**ClassValuePicker** (implements weka.gui.beans.BeanCommon, weka.gui.beans.DataSourceListener, weka.gui.beans.EventConstraints, java.io.Serializable, weka.gui.beans.StructureProducer, weka.gui.beans.Visible)- weka.gui.beans.**ClassValuePickerCustomizer** (implements java.beans.Customizer, weka.gui.beans.CustomizerCloseRequester, weka.gui.beans.CustomizerClosingListener)- weka.gui.beans.**Clusterer** (implements weka.gui.beans.BeanCommon, weka.gui.beans.EventConstraints, weka.gui.beans.TestSetListener, weka.gui.beans.TrainingSetListener, weka.gui.beans.UserRequestAcceptor, weka.gui.beans.Visible, weka.gui.beans.WekaWrapper)- weka.gui.beans.**ClustererCustomizer** (implements java.beans.Customizer, weka.gui.beans.CustomizerCloseRequester)- weka.gui.explorer.**ClustererPanel** (implements weka.gui.explorer.Explorer.CapabilitiesFilterChangeListener, weka.gui.explorer.Explorer.ExplorerPanel, weka.gui.explorer.Explorer.LogHandler)- weka.gui.sql.**ConnectionPanel** (implements javax.swing.event.CaretListener)- weka.gui.beans.**CostBenefitAnalysis** (implements weka.gui.beans.BeanCommon, java.beans.beancontext.BeanContextChild, java.io.Serializable, weka.gui.beans.ThresholdDataListener, weka.gui.beans.UserRequestAcceptor, weka.gui.beans.Visible)- weka.gui.beans.**CrossValidationFoldMakerCustomizer** (implements java.beans.Customizer)- weka.gui.explorer.**DataGeneratorPanel**- weka.gui.experiment.**DatasetListPanel** (implements java.awt.event.ActionListener)- weka.gui.beans.**DataVisualizer** (implements java.beans.beancontext.BeanContextChild, weka.gui.beans.DataSourceListener, java.io.Serializable, weka.gui.beans.TestSetListener, weka.gui.beans.TrainingSetListener, weka.gui.beans.UserRequestAcceptor, weka.gui.beans.Visible)
                                                                                                                                                                                                        - weka.gui.beans.**AttributeSummarizer**- weka.gui.beans.**ScatterPlotMatrix**
                                                                                                                                                                                                        - weka.gui.experiment.**DistributeExperimentPanel**- weka.gui.experiment.**Experimenter**- weka.gui.explorer.**Explorer**- weka.gui.beans.**Filter** (implements weka.gui.beans.BeanCommon, weka.gui.beans.DataSource, weka.gui.beans.DataSourceListener, weka.gui.beans.EventConstraints, weka.gui.beans.InstanceListener, java.io.Serializable, weka.gui.beans.TestSetListener, weka.gui.beans.TestSetProducer, weka.gui.beans.TrainingSetListener, weka.gui.beans.TrainingSetProducer, weka.gui.beans.UserRequestAcceptor, weka.gui.beans.Visible, weka.gui.beans.WekaWrapper)- weka.gui.beans.**FilterCustomizer** (implements java.beans.Customizer, weka.gui.beans.CustomizerCloseRequester)- weka.gui.experiment.**GeneratorPropertyIteratorPanel** (implements java.awt.event.ActionListener)- weka.gui.**GenericArrayEditor** (implements java.beans.PropertyEditor)- weka.gui.**GenericObjectEditor.GOEPanel**- weka.gui.beans.**GraphViewer** (implements java.beans.beancontext.BeanContextChild, weka.gui.beans.GraphListener, java.io.Serializable, weka.gui.beans.UserRequestAcceptor, weka.gui.beans.Visible)- weka.gui.graphvisualizer.**GraphVisualizer** (implements weka.gui.graphvisualizer.GraphConstants, weka.gui.graphvisualizer.LayoutCompleteEventListener)- weka.classifiers.bayes.net.**GUI** (implements weka.gui.graphvisualizer.LayoutCompleteEventListener)- weka.gui.experiment.**HostListPanel** (implements java.awt.event.ActionListener)- weka.gui.beans.**IncrementalClassifierEvaluatorCustomizer** (implements java.beans.Customizer)- weka.gui.sql.**InfoPanel**- weka.gui.streams.**InstanceCounter** (implements weka.gui.streams.InstanceListener)- weka.gui.streams.**InstanceLoader** (implements java.awt.event.ActionListener, weka.gui.streams.InstanceProducer)- weka.gui.**InstancesSummaryPanel**- weka.gui.beans.**InstanceStreamToBatchMaker** (implements weka.gui.beans.BeanCommon, weka.gui.beans.DataSource, weka.gui.beans.EventConstraints, weka.gui.beans.InstanceListener, weka.gui.beans.Visible)- weka.gui.streams.**InstanceTable** (implements weka.gui.streams.InstanceListener)- weka.gui.streams.**InstanceViewer** (implements weka.gui.streams.InstanceListener)- weka.gui.beans.**KnowledgeFlowApp** (implements java.beans.PropertyChangeListener)- weka.gui.beans.**LoaderCustomizer** (implements java.beans.Customizer, weka.gui.beans.CustomizerCloseRequester)- weka.gui.**LogPanel** (implements weka.gui.Logger, weka.gui.TaskLogger)- weka.gui.beans.**LogPanel** (implements weka.gui.Logger)- weka.gui.visualize.**MatrixPanel**
                                                                                                                                                                                                                                                          - weka.gui.explorer.**VisualizePanel** (implements weka.gui.explorer.Explorer.ExplorerPanel)
                                                                                                                                                                                                                                                          - weka.gui.**MemoryUsagePanel**- weka.gui.beans.**MetaBean** (implements weka.gui.beans.BeanCommon, weka.gui.beans.EventConstraints, java.io.Serializable, weka.gui.beans.UserRequestAcceptor, weka.gui.beans.Visible)- weka.gui.beans.**ModelPerformanceChart** (implements java.beans.beancontext.BeanContextChild, java.io.Serializable, weka.gui.beans.ThresholdDataListener, weka.gui.beans.UserRequestAcceptor, weka.gui.beans.Visible, weka.gui.beans.VisualizableErrorListener)- weka.gui.visualize.**Plot2D**- weka.gui.beans.**PredictionAppender** (implements weka.gui.beans.BatchClassifierListener, weka.gui.beans.BatchClustererListener, weka.gui.beans.BeanCommon, weka.gui.beans.DataSource, weka.gui.beans.EventConstraints, weka.gui.beans.IncrementalClassifierListener, java.io.Serializable, weka.gui.beans.TestSetProducer, weka.gui.beans.TrainingSetProducer, weka.gui.beans.Visible)- weka.gui.beans.**PredictionAppenderCustomizer** (implements java.beans.Customizer)- weka.gui.explorer.**PreprocessPanel** (implements weka.gui.explorer.Explorer.CapabilitiesFilterChangeListener, weka.gui.explorer.Explorer.ExplorerPanel, weka.gui.explorer.Explorer.LogHandler)- weka.gui.visualize.**PrintablePanel** (implements weka.gui.visualize.PrintableHandler)
                                                                                                                                                                                                                                                                          - weka.gui.**AttributeVisualizationPanel**- weka.gui.hierarchyvisualizer.**HierarchyVisualizer** (implements java.awt.event.ComponentListener)- weka.gui.treevisualizer.**TreeVisualizer** (implements java.awt.event.ActionListener, java.awt.event.ItemListener, java.awt.event.MouseListener, java.awt.event.MouseMotionListener)- weka.gui.visualize.**VisualizePanel**
                                                                                                                                                                                                                                                                                  - weka.gui.visualize.**ThresholdVisualizePanel**
                                                                                                                                                                                                                                                                          - weka.gui.**PropertyPanel**- weka.gui.**PropertySheetPanel** (implements java.beans.PropertyChangeListener)- weka.gui.sql.**QueryPanel** (implements javax.swing.event.CaretListener, weka.gui.sql.event.ConnectionListener)- weka.gui.**ResultHistoryPanel**- weka.gui.sql.**ResultPanel** (implements javax.swing.event.ChangeListener, weka.gui.sql.event.QueryExecuteListener)- weka.gui.experiment.**ResultsPanel**- weka.gui.experiment.**RunNumberPanel**- weka.gui.experiment.**RunPanel** (implements java.awt.event.ActionListener)- weka.gui.beans.**SaverCustomizer** (implements java.beans.Customizer, weka.gui.beans.CustomizerCloseRequester)- weka.gui.beans.**SerializedModelSaver** (implements weka.gui.beans.BatchClassifierListener, weka.gui.beans.BatchClustererListener, weka.gui.beans.BeanCommon, weka.core.EnvironmentHandler, weka.gui.beans.IncrementalClassifierListener, java.io.Serializable, weka.gui.beans.Visible)- weka.gui.beans.**SerializedModelSaverCustomizer** (implements java.beans.Customizer, weka.gui.beans.CustomizerCloseRequester)- weka.gui.**SetInstancesPanel**- weka.gui.experiment.**SetupModePanel**- weka.gui.experiment.**SetupPanel**- weka.gui.**SimpleCLIPanel** (implements java.awt.event.ActionListener)- weka.gui.experiment.**SimpleSetupPanel**- weka.gui.sql.**SqlViewer** (implements weka.gui.sql.event.ConnectionListener, weka.gui.sql.event.HistoryChangedListener, weka.gui.sql.event.QueryExecuteListener, weka.gui.sql.event.ResultChangedListener)- weka.gui.beans.**StripChart** (implements weka.gui.beans.BeanCommon, weka.gui.beans.ChartListener, weka.gui.beans.InstanceListener, weka.gui.beans.UserRequestAcceptor, weka.gui.beans.Visible)- weka.gui.beans.**StripChartCustomizer** (implements java.beans.Customizer)- weka.gui.beans.**TextViewer** (implements weka.gui.beans.BeanCommon, java.beans.beancontext.BeanContextChild, weka.gui.beans.DataSourceListener, weka.gui.beans.EventConstraints, weka.gui.beans.TestSetListener, weka.gui.beans.TextListener, weka.gui.beans.TrainingSetListener, weka.gui.beans.UserRequestAcceptor, weka.gui.beans.Visible)- weka.gui.beans.**TrainTestSplitMakerCustomizer** (implements java.beans.Customizer)- weka.gui.**WekaTaskMonitor** (implements weka.gui.TaskLogger)
                                                                                                                                - javax.swing.JPopupMenu (implements javax.accessibility.Accessible, javax.swing.MenuElement)
                                                                                                                                  - weka.gui.**GenericObjectEditor.JTreePopupMenu**
                                                                                                                                  - javax.swing.JScrollPane (implements javax.accessibility.Accessible, javax.swing.ScrollPaneConstants)
                                                                                                                                    - weka.gui.visualize.**AttributePanel**- weka.gui.visualize.**LegendPanel**
                                                                                                                                    - javax.swing.JTable (implements javax.accessibility.Accessible, javax.swing.event.CellEditorListener, javax.swing.event.ListSelectionListener, javax.swing.event.RowSorterListener, javax.swing.Scrollable, javax.swing.event.TableColumnModelListener, javax.swing.event.TableModelListener)
                                                                                                                                      - weka.gui.arffviewer.**ArffTable**- weka.gui.sql.**ResultSetTable**
                                                                                                                - java.awt.Panel (implements javax.accessibility.Accessible)
                                                                                                                  - weka.gui.streams.**InstanceSavePanel** (implements weka.gui.streams.InstanceListener)
                                                                                                                  - java.awt.Window (implements javax.accessibility.Accessible)
                                                                                                                    - java.awt.Dialog
                                                                                                                      - javax.swing.JDialog (implements javax.accessibility.Accessible, javax.swing.RootPaneContainer, javax.swing.WindowConstants)
                                                                                                                        - weka.gui.**DatabaseConnectionDialog**- weka.gui.**GenericObjectEditor.CapabilitiesFilterDialog**- weka.gui.**ListSelectorDialog**- weka.gui.experiment.**OutputFormatDialog**- weka.gui.**PropertyDialog**- weka.gui.**PropertySelectorDialog**- weka.gui.sql.**SqlViewerDialog** (implements weka.gui.sql.event.ResultChangedListener)- weka.gui.**ViewerDialog** (implements javax.swing.event.ChangeListener)
                                                                                                                      - java.awt.Frame (implements java.awt.MenuContainer)
                                                                                                                        - javax.swing.JFrame (implements javax.accessibility.Accessible, javax.swing.RootPaneContainer, javax.swing.WindowConstants)
                                                                                                                          - weka.gui.arffviewer.**ArffViewer** (implements java.awt.event.WindowListener)- weka.gui.**GUIChooser**- weka.gui.**GUIChooser.ChildFrameSDI**- weka.gui.**LogWindow** (implements javax.swing.event.CaretListener, javax.swing.event.ChangeListener)- weka.gui.**Main** (implements weka.core.OptionHandler)- weka.gui.**Main.ChildFrameSDI**
                                                                                                                          - weka.gui.**SimpleCLI**
                                                                                                                        - weka.gui.**SplashWindow**
                                                                                                            - weka.gui.**ComponentHelper**- weka.attributeSelection.**ConsistencySubsetEval.hashKey** (implements weka.core.RevisionHandler, java.io.Serializable)- weka.core.**ContingencyTables** (implements weka.core.RevisionHandler)- weka.core.converters.**ConverterUtils** (implements weka.core.RevisionHandler, java.io.Serializable)- weka.core.converters.**ConverterUtils.DataSink** (implements weka.core.RevisionHandler, java.io.Serializable)- weka.core.converters.**ConverterUtils.DataSource** (implements weka.core.RevisionHandler, java.io.Serializable)- weka.core.**Copyright**- weka.classifiers.trees.m5.**CorrelationSplitInfo** (implements java.lang.Cloneable, weka.core.RevisionHandler, java.io.Serializable, weka.classifiers.trees.m5.SplitEvaluate)- weka.classifiers.evaluation.**CostCurve** (implements weka.core.RevisionHandler)- weka.classifiers.**CostMatrix** (implements weka.core.RevisionHandler, java.io.Serializable)- weka.gui.**CostMatrixEditor** (implements java.beans.PropertyEditor)- weka.core.neighboursearch.**CoverTree.CoverTreeNode** (implements weka.core.RevisionHandler, java.io.Serializable)- weka.experiment.**CrossValidationResultProducer** (implements weka.core.AdditionalMeasureProducer, weka.core.OptionHandler, weka.experiment.ResultProducer, weka.core.RevisionHandler)- weka.experiment.**CSVResultListener** (implements weka.core.OptionHandler, weka.experiment.ResultListener, weka.core.RevisionHandler)
                                                                                                                                        - weka.experiment.**InstancesResultListener**
                                                                                                                                        - weka.experiment.**DatabaseUtils** (implements weka.core.RevisionHandler, java.io.Serializable)
                                                                                                                                          - weka.core.converters.**DatabaseConnection**- weka.experiment.**DatabaseResultListener** (implements weka.experiment.ResultListener)
                                                                                                                                              - weka.experiment.**DatabaseResultProducer** (implements weka.core.AdditionalMeasureProducer, weka.core.OptionHandler, weka.experiment.ResultProducer)
                                                                                                                                              - weka.gui.sql.**DbUtils**- weka.experiment.**InstanceQuery** (implements weka.core.OptionHandler)
                                                                                                                                          - weka.datagenerators.**DataGenerator** (implements weka.core.OptionHandler, weka.core.Randomizable, weka.core.RevisionHandler, java.io.Serializable)
                                                                                                                                            - weka.datagenerators.**ClassificationGenerator**
                                                                                                                                              - weka.datagenerators.classifiers.classification.**Agrawal** (implements weka.core.TechnicalInformationHandler)- weka.datagenerators.classifiers.classification.**BayesNet**- weka.datagenerators.classifiers.classification.**LED24** (implements weka.core.TechnicalInformationHandler)- weka.datagenerators.classifiers.classification.**RandomRBF**- weka.datagenerators.classifiers.classification.**RDG1**
                                                                                                                                              - weka.datagenerators.**ClusterGenerator**
                                                                                                                                                - weka.datagenerators.clusterers.**BIRCHCluster** (implements weka.core.TechnicalInformationHandler)- weka.datagenerators.clusterers.**SubspaceCluster**
                                                                                                                                                - weka.datagenerators.**RegressionGenerator**
                                                                                                                                                  - weka.datagenerators.classifiers.regression.**MexicanHat**
                                                                                                                                                    - weka.datagenerators.classifiers.regression.**Expression**
                                                                                                                                            - weka.estimators.**DDConditionalEstimator** (implements weka.estimators.ConditionalEstimator)- weka.core.**Debug** (implements weka.core.RevisionHandler, java.io.Serializable)- weka.core.**Debug.Clock** (implements weka.core.RevisionHandler, java.io.Serializable)- weka.core.**Debug.DBO** (implements weka.core.RevisionHandler, java.io.Serializable)- weka.core.**Debug.Log** (implements weka.core.RevisionHandler, java.io.Serializable)- weka.core.**Debug.SimpleLog** (implements weka.core.RevisionHandler, java.io.Serializable)- weka.core.**Debug.Timestamp** (implements weka.core.RevisionHandler, java.io.Serializable)- weka.classifiers.rules.**DecisionTableHashKey** (implements weka.core.RevisionHandler, java.io.Serializable)- javax.swing.tree.DefaultMutableTreeNode (implements java.lang.Cloneable, javax.swing.tree.MutableTreeNode, java.io.Serializable)
                                                                                                                                                              - weka.gui.**GenericObjectEditor.GOETreeNode**- weka.core.**Trie.TrieNode** (implements weka.core.RevisionHandler)
                                                                                                                                                              - weka.experiment.**DensityBasedClustererSplitEvaluator** (implements weka.core.AdditionalMeasureProducer, weka.core.OptionHandler, weka.core.RevisionHandler, weka.experiment.SplitEvaluator)- java.util.Dictionary<K,V>
                                                                                                                                                                  - java.util.Hashtable<K,V> (implements java.lang.Cloneable, java.util.Map<K,V>, java.io.Serializable)
                                                                                                                                                                    - java.util.Properties
                                                                                                                                                                      - weka.core.**ProtectedProperties** (implements weka.core.RevisionHandler)
                                                                                                                                                                  - weka.classifiers.functions.pace.**DiscreteFunction** (implements weka.core.RevisionHandler)- weka.classifiers.trees.j48.**Distribution** (implements java.lang.Cloneable, weka.core.RevisionHandler, java.io.Serializable)- weka.estimators.**DKConditionalEstimator** (implements weka.estimators.ConditionalEstimator)- weka.classifiers.bayes.**DMNBtext.DNBBinary** (implements java.io.Serializable)- weka.estimators.**DNConditionalEstimator** (implements weka.estimators.ConditionalEstimator)- weka.gui.graphvisualizer.**DotParser** (implements weka.gui.graphvisualizer.GraphConstants)- weka.core.matrix.**DoubleVector** (implements java.lang.Cloneable, weka.core.RevisionHandler)- weka.gui.treevisualizer.**Edge**- weka.core.matrix.**EigenvalueDecomposition** (implements weka.core.RevisionHandler, java.io.Serializable)- weka.associations.gsp.**Element** (implements java.lang.Cloneable, weka.core.RevisionHandler, java.io.Serializable)- weka.core.**Environment** (implements weka.core.RevisionHandler)- weka.clusterers.forOPTICSAndDBScan.Utils.**EpsilonRange\_ListElement** (implements weka.core.RevisionHandler)- weka.estimators.**Estimator** (implements weka.core.CapabilitiesHandler, java.lang.Cloneable, weka.core.OptionHandler, weka.core.RevisionHandler, java.io.Serializable)
                                                                                                                                                                                            - weka.estimators.**DiscreteEstimator** (implements weka.estimators.IncrementalEstimator)- weka.classifiers.bayes.net.estimate.**DiscreteEstimatorBayes** (implements weka.classifiers.bayes.net.search.local.Scoreable)
                                                                                                                                                                                                - weka.classifiers.bayes.net.estimate.**DiscreteEstimatorFullBayes**
                                                                                                                                                                                                - weka.estimators.**KernelEstimator** (implements weka.estimators.IncrementalEstimator)- weka.estimators.**MahalanobisEstimator** (implements weka.estimators.IncrementalEstimator)- weka.estimators.**NormalEstimator** (implements weka.estimators.IncrementalEstimator)- weka.estimators.**PoissonEstimator** (implements weka.estimators.IncrementalEstimator)
                                                                                                                                                                                            - weka.estimators.**EstimatorUtils** (implements weka.core.RevisionHandler)- weka.clusterers.forOPTICSAndDBScan.DataObjects.**EuclidianDataObject** (implements weka.clusterers.forOPTICSAndDBScan.DataObjects.DataObject, weka.core.RevisionHandler, java.io.Serializable)- weka.classifiers.**Evaluation** (implements weka.core.RevisionHandler, weka.core.Summarizable)- weka.classifiers.evaluation.**EvaluationUtils** (implements weka.core.RevisionHandler)- java.util.EventObject (implements java.io.Serializable)
                                                                                                                                                                                                      - weka.gui.beans.**BatchClassifierEvent**- weka.gui.beans.**BatchClustererEvent**- javax.swing.event.ChangeEvent
                                                                                                                                                                                                            - weka.gui.explorer.**Explorer.CapabilitiesFilterChangeEvent**
                                                                                                                                                                                                            - weka.gui.beans.**ChartEvent**- weka.gui.sql.event.**ConnectionEvent**- weka.gui.beans.**DataSetEvent**- weka.gui.beans.**GraphEvent**- weka.gui.sql.event.**HistoryChangedEvent**- weka.gui.beans.**IncrementalClassifierEvent**- weka.gui.beans.**InstanceEvent**- weka.gui.streams.**InstanceEvent**- weka.gui.graphvisualizer.**LayoutCompleteEvent**- weka.gui.sql.event.**QueryExecuteEvent**- weka.gui.sql.event.**ResultChangedEvent**- weka.gui.beans.**TestSetEvent**- weka.gui.beans.**TextEvent**- weka.gui.beans.**ThresholdDataEvent**- weka.gui.beans.**TrainingSetEvent**- weka.gui.beans.**VisualizableErrorEvent**
                                                                                                                                                                                                      - weka.experiment.**Experiment** (implements weka.core.OptionHandler, weka.core.RevisionHandler, java.io.Serializable)
                                                                                                                                                                                                        - weka.experiment.**RemoteExperiment**
                                                                                                                                                                                                        - weka.gui.experiment.**ExperimenterDefaults** (implements java.io.Serializable)- weka.gui.explorer.**ExplorerDefaults** (implements java.io.Serializable)- weka.core.pmml.**Expression** (implements java.io.Serializable)
                                                                                                                                                                                                              - weka.core.pmml.**Constant**- weka.core.pmml.**Discretize**- weka.core.pmml.**FieldRef**- weka.core.pmml.**NormContinuous**- weka.core.pmml.**NormDiscrete**
                                                                                                                                                                                                              - weka.core.**FastVector** (implements weka.core.Copyable, weka.core.RevisionHandler, java.io.Serializable)
                                                                                                                                                                                                                - weka.attributeSelection.**BestFirst.LinkedList2**- weka.attributeSelection.**LFSMethods.LinkedList2**
                                                                                                                                                                                                                - weka.core.**FastVector.FastVectorEnumeration** (implements java.util.Enumeration<E>, weka.core.RevisionHandler)- weka.core.pmml.**FieldMetaInfo** (implements java.io.Serializable)
                                                                                                                                                                                                                    - weka.core.pmml.**DerivedFieldMetaInfo** (implements java.io.Serializable)- weka.core.pmml.**MiningFieldMetaInfo** (implements java.io.Serializable)- weka.core.pmml.**TargetMetaInfo** (implements java.io.Serializable)
                                                                                                                                                                                                                    - weka.core.pmml.**FieldMetaInfo.Interval** (implements java.io.Serializable)- weka.core.pmml.**FieldMetaInfo.Value** (implements java.io.Serializable)- javax.swing.filechooser.FileFilter
                                                                                                                                                                                                                          - weka.gui.**ExtensionFileFilter** (implements java.io.FilenameFilter, java.io.Serializable)- weka.clusterers.forOPTICSAndDBScan.OPTICS\_GUI.**SERFileFilter** (implements weka.core.RevisionHandler)
                                                                                                                                                                                                                          - weka.filters.**Filter** (implements weka.core.CapabilitiesHandler, weka.core.RevisionHandler, java.io.Serializable)
                                                                                                                                                                                                                            - weka.filters.unsupervised.attribute.**AbstractTimeSeries** (implements weka.core.OptionHandler, weka.filters.UnsupervisedFilter)
                                                                                                                                                                                                                              - weka.filters.unsupervised.attribute.**TimeSeriesTranslate**
                                                                                                                                                                                                                                - weka.filters.unsupervised.attribute.**TimeSeriesDelta**
                                                                                                                                                                                                                              - weka.filters.unsupervised.attribute.**Add** (implements weka.core.OptionHandler, weka.filters.StreamableFilter, weka.filters.UnsupervisedFilter)- weka.filters.unsupervised.attribute.**AddCluster** (implements weka.core.OptionHandler, weka.filters.UnsupervisedFilter)- weka.filters.unsupervised.attribute.**AddExpression** (implements weka.core.OptionHandler, weka.filters.StreamableFilter, weka.filters.UnsupervisedFilter)- weka.filters.unsupervised.attribute.**AddID** (implements weka.core.OptionHandler, weka.filters.UnsupervisedFilter)- weka.filters.unsupervised.attribute.**AddNoise** (implements weka.core.OptionHandler, weka.filters.UnsupervisedFilter)- weka.filters.unsupervised.attribute.**AddValues** (implements weka.core.OptionHandler, weka.filters.StreamableFilter, weka.filters.UnsupervisedFilter)- weka.filters.**AllFilter** (implements weka.filters.Sourcable)- weka.filters.supervised.attribute.**AttributeSelection** (implements weka.core.OptionHandler, weka.filters.SupervisedFilter)- weka.filters.unsupervised.attribute.**ChangeDateFormat** (implements weka.core.OptionHandler, weka.filters.StreamableFilter, weka.filters.UnsupervisedFilter)- weka.filters.supervised.attribute.**ClassOrder** (implements weka.core.OptionHandler, weka.filters.SupervisedFilter)- weka.filters.unsupervised.attribute.**ClusterMembership** (implements weka.core.OptionHandler, weka.filters.UnsupervisedFilter)- weka.filters.unsupervised.attribute.**Copy** (implements weka.core.OptionHandler, weka.filters.StreamableFilter, weka.filters.UnsupervisedFilter)- weka.filters.supervised.attribute.**Discretize** (implements weka.core.OptionHandler, weka.filters.SupervisedFilter, weka.core.TechnicalInformationHandler, weka.core.WeightedInstancesHandler)- weka.filters.unsupervised.attribute.**FirstOrder** (implements weka.core.OptionHandler, weka.filters.StreamableFilter, weka.filters.UnsupervisedFilter)- weka.filters.unsupervised.attribute.**MakeIndicator** (implements weka.core.OptionHandler, weka.filters.StreamableFilter, weka.filters.UnsupervisedFilter)- weka.filters.unsupervised.attribute.**MergeTwoValues** (implements weka.core.OptionHandler, weka.filters.StreamableFilter, weka.filters.UnsupervisedFilter)- weka.filters.unsupervised.attribute.**MultiInstanceToPropositional** (implements weka.core.MultiInstanceCapabilitiesHandler, weka.core.OptionHandler, weka.filters.UnsupervisedFilter)- weka.filters.supervised.attribute.**NominalToBinary** (implements weka.core.OptionHandler, weka.filters.SupervisedFilter, weka.core.TechnicalInformationHandler)- weka.filters.unsupervised.attribute.**NominalToBinary** (implements weka.core.OptionHandler, weka.filters.UnsupervisedFilter)- weka.filters.unsupervised.attribute.**NominalToString** (implements weka.core.OptionHandler, weka.filters.UnsupervisedFilter)- weka.filters.unsupervised.instance.**NonSparseToSparse** (implements weka.filters.StreamableFilter, weka.filters.UnsupervisedFilter)- weka.filters.unsupervised.instance.**Normalize** (implements weka.core.OptionHandler, weka.filters.UnsupervisedFilter)- weka.filters.unsupervised.attribute.**NumericTransform** (implements weka.core.OptionHandler, weka.filters.StreamableFilter, weka.filters.UnsupervisedFilter)- weka.filters.unsupervised.attribute.**Obfuscate** (implements weka.filters.StreamableFilter, weka.filters.UnsupervisedFilter)- weka.filters.unsupervised.attribute.**PotentialClassIgnorer** (implements weka.core.OptionHandler)
                                                                                                                                                                                                                                                                                - weka.filters.unsupervised.attribute.**Center** (implements weka.filters.Sourcable, weka.filters.UnsupervisedFilter)- weka.filters.unsupervised.attribute.**Discretize** (implements weka.filters.UnsupervisedFilter, weka.core.WeightedInstancesHandler)
                                                                                                                                                                                                                                                                                    - weka.filters.unsupervised.attribute.**PKIDiscretize** (implements weka.core.TechnicalInformationHandler)
                                                                                                                                                                                                                                                                                    - weka.filters.unsupervised.attribute.**MathExpression** (implements weka.filters.UnsupervisedFilter)- weka.filters.unsupervised.attribute.**Normalize** (implements weka.core.OptionHandler, weka.filters.Sourcable, weka.filters.UnsupervisedFilter)- weka.filters.unsupervised.attribute.**NumericToBinary** (implements weka.filters.StreamableFilter, weka.filters.UnsupervisedFilter)- weka.filters.unsupervised.attribute.**ReplaceMissingValues** (implements weka.filters.Sourcable, weka.filters.UnsupervisedFilter)- weka.filters.unsupervised.attribute.**Standardize** (implements weka.filters.Sourcable, weka.filters.UnsupervisedFilter)
                                                                                                                                                                                                                                                                                - weka.filters.unsupervised.attribute.**PrincipalComponents** (implements weka.core.OptionHandler, weka.filters.UnsupervisedFilter)- weka.filters.unsupervised.attribute.**PropositionalToMultiInstance** (implements weka.core.OptionHandler, weka.filters.UnsupervisedFilter)- weka.filters.unsupervised.instance.**Randomize** (implements weka.core.OptionHandler, weka.filters.UnsupervisedFilter)- weka.filters.unsupervised.attribute.**RandomProjection** (implements weka.core.OptionHandler, weka.core.TechnicalInformationHandler, weka.filters.UnsupervisedFilter)- weka.filters.unsupervised.attribute.**Remove** (implements weka.core.OptionHandler, weka.filters.StreamableFilter, weka.filters.UnsupervisedFilter)- weka.filters.unsupervised.instance.**RemoveFolds** (implements weka.core.OptionHandler, weka.filters.UnsupervisedFilter)- weka.filters.unsupervised.instance.**RemoveFrequentValues** (implements weka.core.OptionHandler, weka.filters.UnsupervisedFilter)- weka.filters.unsupervised.instance.**RemoveMisclassified** (implements weka.core.OptionHandler, weka.filters.UnsupervisedFilter)- weka.filters.unsupervised.instance.**RemovePercentage** (implements weka.core.OptionHandler, weka.filters.UnsupervisedFilter)- weka.filters.unsupervised.instance.**RemoveRange** (implements weka.core.OptionHandler, weka.filters.UnsupervisedFilter)- weka.filters.unsupervised.attribute.**RemoveType** (implements weka.core.OptionHandler, weka.filters.StreamableFilter, weka.filters.UnsupervisedFilter)- weka.filters.unsupervised.attribute.**RemoveUseless** (implements weka.core.OptionHandler, weka.filters.UnsupervisedFilter)- weka.filters.unsupervised.instance.**RemoveWithValues** (implements weka.core.OptionHandler, weka.filters.StreamableFilter, weka.filters.UnsupervisedFilter)- weka.filters.unsupervised.attribute.**Reorder** (implements weka.core.OptionHandler, weka.filters.StreamableFilter, weka.filters.UnsupervisedFilter)- weka.filters.supervised.instance.**Resample** (implements weka.core.OptionHandler, weka.filters.SupervisedFilter)- weka.filters.unsupervised.instance.**Resample** (implements weka.core.OptionHandler, weka.filters.UnsupervisedFilter)- weka.filters.unsupervised.instance.**ReservoirSample** (implements weka.core.OptionHandler, weka.filters.StreamableFilter, weka.filters.UnsupervisedFilter)- weka.filters.**SimpleFilter** (implements weka.core.OptionHandler)
                                                                                                                                                                                                                                                                                                                    - weka.filters.**SimpleBatchFilter**
                                                                                                                                                                                                                                                                                                                      - weka.filters.supervised.attribute.**AddClassification**- weka.filters.unsupervised.attribute.**InterquartileRange**- weka.filters.unsupervised.attribute.**KernelFilter** (implements weka.core.TechnicalInformationHandler, weka.filters.UnsupervisedFilter)- weka.filters.unsupervised.attribute.**NumericToNominal**- weka.filters.unsupervised.attribute.**PartitionedMultiFilter**- weka.filters.supervised.attribute.**PLSFilter** (implements weka.filters.SupervisedFilter, weka.core.TechnicalInformationHandler)- weka.filters.unsupervised.attribute.**RELAGGS** (implements weka.core.TechnicalInformationHandler)- weka.filters.unsupervised.instance.**SubsetByExpression**- weka.filters.unsupervised.attribute.**Wavelet** (implements weka.core.TechnicalInformationHandler)
                                                                                                                                                                                                                                                                                                                      - weka.filters.**SimpleStreamFilter** (implements weka.filters.StreamableFilter)
                                                                                                                                                                                                                                                                                                                        - weka.filters.unsupervised.attribute.**ClassAssigner**- weka.filters.**MultiFilter**- weka.filters.unsupervised.attribute.**NumericCleaner**- weka.filters.unsupervised.attribute.**RandomSubset**
                                                                                                                                                                                                                                                                                                                    - weka.filters.supervised.instance.**SMOTE** (implements weka.core.OptionHandler, weka.filters.SupervisedFilter, weka.core.TechnicalInformationHandler)- weka.filters.unsupervised.instance.**SparseToNonSparse** (implements weka.filters.StreamableFilter, weka.filters.UnsupervisedFilter)- weka.filters.supervised.instance.**SpreadSubsample** (implements weka.core.OptionHandler, weka.filters.SupervisedFilter)- weka.filters.supervised.instance.**StratifiedRemoveFolds** (implements weka.core.OptionHandler, weka.filters.SupervisedFilter)- weka.filters.unsupervised.attribute.**StringToNominal** (implements weka.core.OptionHandler, weka.filters.UnsupervisedFilter)- weka.filters.unsupervised.attribute.**StringToWordVector** (implements weka.core.OptionHandler, weka.filters.UnsupervisedFilter)- weka.filters.unsupervised.attribute.**SwapValues** (implements weka.core.OptionHandler, weka.filters.StreamableFilter, weka.filters.UnsupervisedFilter)
                                                                                                                                                                                                                            - weka.core.**FindWithCapabilities** (implements weka.core.CapabilitiesHandler, weka.core.OptionHandler, weka.core.RevisionHandler)- weka.gui.beans.**FlowRunner** (implements weka.core.RevisionHandler)- weka.gui.beans.**FlowRunner.SimpleLogger** (implements weka.gui.Logger)- java.text.Format (implements java.lang.Cloneable, java.io.Serializable)
                                                                                                                                                                                                                                    - java.text.NumberFormat
                                                                                                                                                                                                                                      - java.text.DecimalFormat
                                                                                                                                                                                                                                        - weka.core.matrix.**ExponentialFormat** (implements weka.core.RevisionHandler)- weka.core.matrix.**FlexibleDecimalFormat** (implements weka.core.RevisionHandler)- weka.core.matrix.**FloatingPointFormat** (implements weka.core.RevisionHandler)
                                                                                                                                                                                                                                    - weka.associations.**FPGrowth.AssociationRule** (implements java.lang.Comparable<T>, java.io.Serializable)- weka.associations.**FPGrowth.BinaryItem** (implements java.lang.Comparable<T>, java.io.Serializable)- weka.core.pmml.**Function** (implements java.io.Serializable)
                                                                                                                                                                                                                                          - weka.core.pmml.**BuiltInArithmetic**- weka.core.pmml.**BuiltInMath**- weka.core.pmml.**BuiltInString**- weka.core.pmml.**DefineFunction**
                                                                                                                                                                                                                                          - weka.gui.**GenericObjectEditor** (implements weka.gui.CustomPanelSupplier, java.beans.PropertyEditor)- weka.gui.**GenericPropertiesCreator**- weka.gui.graphvisualizer.**GraphEdge**- java.awt.Graphics
                                                                                                                                                                                                                                                  - java.awt.Graphics2D
                                                                                                                                                                                                                                                    - weka.gui.visualize.**PostscriptGraphics**
                                                                                                                                                                                                                                                  - weka.gui.graphvisualizer.**GraphNode** (implements weka.gui.graphvisualizer.GraphConstants)- weka.gui.graphvisualizer.**HierarchicalBCEngine** (implements weka.gui.graphvisualizer.GraphConstants, weka.gui.graphvisualizer.LayoutEngine)- weka.gui.**HierarchyPropertyParser** (implements java.io.Serializable)- weka.classifiers.trees.m5.**Impurity** (implements weka.core.RevisionHandler)- weka.core.**Instance** (implements weka.core.Copyable, weka.core.RevisionHandler, java.io.Serializable)
                                                                                                                                                                                                                                                            - weka.associations.tertius.**IndividualInstance**- weka.core.**SparseInstance**
                                                                                                                                                                                                                                                                - weka.core.**BinarySparseInstance**
                                                                                                                                                                                                                                                            - weka.core.**InstanceComparator** (implements java.util.Comparator<T>, weka.core.RevisionHandler, java.io.Serializable)- weka.gui.streams.**InstanceJoiner** (implements weka.gui.streams.InstanceProducer, weka.gui.streams.SerialInstanceListener, java.io.Serializable)- weka.core.**Instances** (implements weka.core.RevisionHandler, java.io.Serializable)
                                                                                                                                                                                                                                                                  - weka.associations.tertius.**IndividualInstances**- weka.classifiers.trees.adtree.**ReferenceInstances**
                                                                                                                                                                                                                                                                  - weka.core.matrix.**IntVector** (implements java.lang.Cloneable, weka.core.RevisionHandler)- weka.associations.**ItemSet** (implements weka.core.RevisionHandler, java.io.Serializable)
                                                                                                                                                                                                                                                                      - weka.associations.**AprioriItemSet** (implements weka.core.RevisionHandler, java.io.Serializable)- weka.associations.**LabeledItemSet** (implements weka.core.RevisionHandler, java.io.Serializable)
                                                                                                                                                                                                                                                                      - weka.core.**Javadoc** (implements weka.core.OptionHandler, weka.core.RevisionHandler)
                                                                                                                                                                                                                                                                        - weka.core.**AllJavadoc**- weka.core.**GlobalInfoJavadoc**- weka.core.**OptionHandlerJavadoc**- weka.core.**TechnicalInformationHandlerJavadoc**
                                                                                                                                                                                                                                                                        - weka.gui.visualize.**JComponentWriter**
                                                                                                                                                                                                                                                                          - weka.gui.visualize.**BMPWriter**- weka.gui.visualize.**JPEGWriter**- weka.gui.visualize.**PNGWriter**- weka.gui.visualize.**PostscriptWriter**
                                                                                                                                                                                                                                                                          - weka.gui.**JListHelper**- weka.gui.**JTableHelper**- weka.core.**Jython** (implements weka.core.RevisionHandler, java.io.Serializable)- weka.estimators.**KDConditionalEstimator** (implements weka.estimators.ConditionalEstimator)- weka.gui.boundaryvisualizer.**KDDataGenerator** (implements weka.gui.boundaryvisualizer.DataGenerator, java.io.Serializable)- weka.core.neighboursearch.kdtrees.**KDTreeNode** (implements weka.core.RevisionHandler, java.io.Serializable)- weka.core.neighboursearch.kdtrees.**KDTreeNodeSplitter** (implements weka.core.OptionHandler, weka.core.RevisionHandler, java.io.Serializable)
                                                                                                                                                                                                                                                                                        - weka.core.neighboursearch.kdtrees.**KMeansInpiredMethod** (implements weka.core.TechnicalInformationHandler)- weka.core.neighboursearch.kdtrees.**MedianOfWidestDimension** (implements weka.core.TechnicalInformationHandler)- weka.core.neighboursearch.kdtrees.**MidPointOfWidestDimension** (implements weka.core.TechnicalInformationHandler)- weka.core.neighboursearch.kdtrees.**SlidingMidPointOfWidestSide** (implements weka.core.TechnicalInformationHandler)
                                                                                                                                                                                                                                                                                        - weka.classifiers.functions.supportVector.**Kernel** (implements weka.core.CapabilitiesHandler, weka.core.OptionHandler, weka.core.RevisionHandler, java.io.Serializable)
                                                                                                                                                                                                                                                                                          - weka.classifiers.functions.supportVector.**CachedKernel**
                                                                                                                                                                                                                                                                                            - weka.classifiers.functions.supportVector.**PolyKernel**
                                                                                                                                                                                                                                                                                              - weka.classifiers.mi.supportVector.**MIPolyKernel** (implements weka.core.MultiInstanceCapabilitiesHandler)- weka.classifiers.functions.supportVector.**NormalizedPolyKernel**
                                                                                                                                                                                                                                                                                              - weka.classifiers.functions.supportVector.**Puk** (implements weka.core.TechnicalInformationHandler)- weka.classifiers.functions.supportVector.**RBFKernel**
                                                                                                                                                                                                                                                                                                  - weka.classifiers.mi.supportVector.**MIRBFKernel** (implements weka.core.MultiInstanceCapabilitiesHandler)
                                                                                                                                                                                                                                                                                            - weka.classifiers.functions.supportVector.**PrecomputedKernelMatrixKernel**- weka.classifiers.functions.supportVector.**StringKernel** (implements weka.core.TechnicalInformationHandler)
                                                                                                                                                                                                                                                                                          - weka.classifiers.functions.supportVector.**KernelEvaluation** (implements weka.core.RevisionHandler)- java.awt.event.KeyAdapter (implements java.awt.event.KeyListener)
                                                                                                                                                                                                                                                                                              - weka.gui.**ResultHistoryPanel.RKeyAdapter** (implements java.io.Serializable)
                                                                                                                                                                                                                                                                                              - weka.estimators.**KKConditionalEstimator** (implements weka.estimators.ConditionalEstimator)- weka.gui.beans.**KnowledgeFlow**- weka.core.xml.**KOML** (implements weka.core.RevisionHandler)- weka.classifiers.lazy.kstar.**KStarCache** (implements weka.core.RevisionHandler, java.io.Serializable)- weka.classifiers.lazy.kstar.**KStarCache.CacheTable** (implements weka.core.RevisionHandler, java.io.Serializable)- weka.classifiers.lazy.kstar.**KStarCache.TableEntry** (implements weka.core.RevisionHandler, java.io.Serializable)- weka.classifiers.lazy.kstar.**KStarNominalAttribute** (implements weka.classifiers.lazy.kstar.KStarConstants, weka.core.RevisionHandler)- weka.classifiers.lazy.kstar.**KStarNumericAttribute** (implements weka.classifiers.lazy.kstar.KStarConstants, weka.core.RevisionHandler)- weka.classifiers.lazy.kstar.**KStarWrapper** (implements weka.core.RevisionHandler)- weka.classifiers.lazy.**LBR.Indexes** (implements weka.core.RevisionHandler, java.io.Serializable)- weka.experiment.**LearningRateResultProducer** (implements weka.core.AdditionalMeasureProducer, weka.core.OptionHandler, weka.experiment.ResultListener, weka.experiment.ResultProducer, weka.core.RevisionHandler)- weka.attributeSelection.**LFSMethods** (implements weka.core.RevisionHandler)- weka.attributeSelection.**LFSMethods.Link2** (implements weka.core.RevisionHandler, java.io.Serializable)- weka.core.matrix.**LinearRegression** (implements weka.core.RevisionHandler)- weka.classifiers.functions.neural.**LinearUnit** (implements weka.classifiers.functions.neural.NeuralMethod, weka.core.RevisionHandler)- weka.core.**ListOptions** (implements weka.core.OptionHandler, weka.core.RevisionHandler)- weka.associations.tertius.**Literal** (implements weka.core.RevisionHandler, java.io.Serializable)
                                                                                                                                                                                                                                                                                                                                - weka.associations.tertius.**AttributeValueLiteral**
                                                                                                                                                                                                                                                                                                                                  - weka.associations.tertius.**IndividualLiteral**
                                                                                                                                                                                                                                                                                                                                - weka.associations.tertius.**LiteralSet** (implements java.lang.Cloneable, weka.core.RevisionHandler, java.io.Serializable)
                                                                                                                                                                                                                                                                                                                                  - weka.associations.tertius.**Body**- weka.associations.tertius.**Head**
                                                                                                                                                                                                                                                                                                                                  - weka.gui.**Loader**- weka.core.logging.**Logger** (implements weka.core.RevisionHandler)
                                                                                                                                                                                                                                                                                                                                      - weka.core.logging.**ConsoleLogger**
                                                                                                                                                                                                                                                                                                                                        - weka.core.logging.**FileLogger**
                                                                                                                                                                                                                                                                                                                                          - weka.core.logging.**OutputLogger**
                                                                                                                                                                                                                                                                                                                                      - weka.gui.**LookAndFeel**- weka.core.stemmers.**LovinsStemmer** (implements weka.core.stemmers.Stemmer, weka.core.TechnicalInformationHandler)
                                                                                                                                                                                                                                                                                                                                          - weka.core.stemmers.**IteratedLovinsStemmer**
                                                                                                                                                                                                                                                                                                                                          - java\_cup.runtime.lr\_parser
                                                                                                                                                                                                                                                                                                                                            - weka.core.mathematicalexpression.**Parser**- weka.filters.unsupervised.instance.subsetbyexpression.**Parser**
                                                                                                                                                                                                                                                                                                                                            - weka.core.matrix.**LUDecomposition** (implements weka.core.RevisionHandler, java.io.Serializable)- weka.classifiers.rules.part.**MakeDecList** (implements weka.core.CapabilitiesHandler, weka.core.RevisionHandler, java.io.Serializable)- weka.clusterers.forOPTICSAndDBScan.DataObjects.**ManhattanDataObject** (implements weka.clusterers.forOPTICSAndDBScan.DataObjects.DataObject, weka.core.RevisionHandler, java.io.Serializable)- weka.core.pmml.**MappingInfo** (implements java.io.Serializable)- weka.classifiers.bayes.net.**MarginCalculator** (implements weka.core.RevisionHandler, java.io.Serializable)- weka.classifiers.bayes.net.**MarginCalculator.JunctionTreeNode** (implements weka.core.RevisionHandler, java.io.Serializable)- weka.classifiers.bayes.net.**MarginCalculator.JunctionTreeSeparator** (implements weka.core.RevisionHandler, java.io.Serializable)- weka.classifiers.evaluation.**MarginCurve** (implements weka.core.RevisionHandler)- weka.core.**MathematicalExpression** (implements weka.core.RevisionHandler)- weka.core.matrix.**Maths** (implements weka.core.RevisionHandler)- weka.core.**Matrix** (implements java.lang.Cloneable, weka.core.RevisionHandler, java.io.Serializable)
                                                                                                                                                                                                                                                                                                                                                                  - weka.classifiers.evaluation.**ConfusionMatrix**
                                                                                                                                                                                                                                                                                                                                                                  - weka.core.matrix.**Matrix** (implements java.lang.Cloneable, weka.core.RevisionHandler, java.io.Serializable)
                                                                                                                                                                                                                                                                                                                                                                    - weka.classifiers.functions.pace.**PaceMatrix**
                                                                                                                                                                                                                                                                                                                                                                    - weka.core.**Memory** (implements weka.core.RevisionHandler)- weka.associations.**Messages**- weka.associations.gsp.**Messages**- weka.gui.**Messages**- weka.gui.arffviewer.**Messages**- weka.gui.beans.**Messages**- weka.gui.beans.xml.**Messages**- weka.gui.boundaryvisualizer.**Messages**- weka.gui.experiment.**Messages**- weka.gui.explorer.**Messages**- weka.gui.graphvisualizer.**Messages**- weka.gui.hierarchyvisualizer.**Messages**- weka.gui.sql.**Messages**- weka.gui.sql.event.**Messages**- weka.gui.streams.**Messages**- weka.gui.treevisualizer.**Messages**- weka.gui.visualize.**Messages**- weka.core.xml.**MethodHandler** (implements weka.core.RevisionHandler)- weka.core.pmml.**MiningSchema** (implements java.io.Serializable)- weka.classifiers.functions.pace.**MixtureDistribution** (implements weka.core.RevisionHandler, weka.core.TechnicalInformationHandler)
                                                                                                                                                                                                                                                                                                                                                                                                            - weka.classifiers.functions.pace.**ChisqMixture**- weka.classifiers.functions.pace.**NormalMixture**
                                                                                                                                                                                                                                                                                                                                                                                                            - weka.classifiers.trees.j48.**ModelSelection** (implements weka.core.RevisionHandler, java.io.Serializable)
                                                                                                                                                                                                                                                                                                                                                                                                              - weka.classifiers.trees.j48.**BinC45ModelSelection**- weka.classifiers.trees.j48.**C45ModelSelection**- weka.classifiers.trees.j48.**NBTreeModelSelection**- weka.classifiers.trees.lmt.**ResidualModelSelection**
                                                                                                                                                                                                                                                                                                                                                                                                              - java.awt.event.MouseAdapter (implements java.awt.event.MouseListener, java.awt.event.MouseMotionListener, java.awt.event.MouseWheelListener)
                                                                                                                                                                                                                                                                                                                                                                                                                - weka.gui.**ResultHistoryPanel.RMouseAdapter** (implements java.io.Serializable)
                                                                                                                                                                                                                                                                                                                                                                                                                - weka.gui.treevisualizer.**NamedColor**- weka.estimators.**NDConditionalEstimator** (implements weka.estimators.ConditionalEstimator)- weka.core.neighboursearch.**NearestNeighbourSearch** (implements weka.core.AdditionalMeasureProducer, weka.core.OptionHandler, weka.core.RevisionHandler, java.io.Serializable)
                                                                                                                                                                                                                                                                                                                                                                                                                      - weka.core.neighboursearch.**BallTree** (implements weka.core.TechnicalInformationHandler)- weka.core.neighboursearch.**CoverTree** (implements weka.core.TechnicalInformationHandler)- weka.core.neighboursearch.**KDTree** (implements weka.core.TechnicalInformationHandler)- weka.core.neighboursearch.**LinearNNSearch**
                                                                                                                                                                                                                                                                                                                                                                                                                      - weka.classifiers.functions.neural.**NeuralConnection** (implements weka.core.RevisionHandler, java.io.Serializable)
                                                                                                                                                                                                                                                                                                                                                                                                                        - weka.classifiers.functions.neural.**NeuralNode**
                                                                                                                                                                                                                                                                                                                                                                                                                        - weka.estimators.**NNConditionalEstimator** (implements weka.estimators.ConditionalEstimator)- weka.gui.treevisualizer.**Node**- weka.classifiers.evaluation.**NominalPrediction** (implements weka.classifiers.evaluation.Prediction, weka.core.RevisionHandler, java.io.Serializable)- weka.core.**NormalizableDistance** (implements weka.core.DistanceFunction, weka.core.OptionHandler, weka.core.RevisionHandler, java.io.Serializable)
                                                                                                                                                                                                                                                                                                                                                                                                                                - weka.core.**AbstractStringDistanceFunction**
                                                                                                                                                                                                                                                                                                                                                                                                                                  - weka.core.**EditDistance**
                                                                                                                                                                                                                                                                                                                                                                                                                                  - weka.core.**ChebyshevDistance** (implements weka.core.TechnicalInformationHandler)- weka.core.**EuclideanDistance** (implements java.lang.Cloneable, weka.core.TechnicalInformationHandler)- weka.core.**ManhattanDistance** (implements weka.core.TechnicalInformationHandler)
                                                                                                                                                                                                                                                                                                                                                                                                                                - weka.core.stemmers.**NullStemmer** (implements weka.core.stemmers.Stemmer)- weka.classifiers.evaluation.**NumericPrediction** (implements weka.classifiers.evaluation.Prediction, weka.core.RevisionHandler, java.io.Serializable)- weka.clusterers.forOPTICSAndDBScan.OPTICS\_GUI.**OPTICS\_Visualizer** (implements weka.core.RevisionHandler)- weka.core.**Optimization** (implements weka.core.RevisionHandler, weka.core.TechnicalInformationHandler)- weka.core.**Option** (implements weka.core.RevisionHandler)- java.io.OutputStream (implements java.io.Closeable, java.io.Flushable)
                                                                                                                                                                                                                                                                                                                                                                                                                                            - java.io.FilterOutputStream
                                                                                                                                                                                                                                                                                                                                                                                                                                              - java.io.PrintStream (implements java.lang.Appendable, java.io.Closeable)
                                                                                                                                                                                                                                                                                                                                                                                                                                                - weka.core.logging.**OutputLogger.OutputPrintStream**- weka.core.**Tee** (implements weka.core.RevisionHandler)
                                                                                                                                                                                                                                                                                                                                                                                                                                            - weka.experiment.**OutputZipper** (implements weka.core.RevisionHandler)- weka.experiment.**PairedStats** (implements weka.core.RevisionHandler)
                                                                                                                                                                                                                                                                                                                                                                                                                                                - weka.experiment.**PairedStatsCorrected**
                                                                                                                                                                                                                                                                                                                                                                                                                                                - weka.experiment.**PairedTTester** (implements weka.core.OptionHandler, weka.core.RevisionHandler, weka.experiment.Tester)
                                                                                                                                                                                                                                                                                                                                                                                                                                                  - weka.experiment.**PairedCorrectedTTester** (implements weka.core.TechnicalInformationHandler)
                                                                                                                                                                                                                                                                                                                                                                                                                                                  - weka.classifiers.bayes.net.**ParentSet** (implements weka.core.RevisionHandler, java.io.Serializable)- weka.core.neighboursearch.**PerformanceStats** (implements weka.core.AdditionalMeasureProducer, weka.core.RevisionHandler, java.io.Serializable)
                                                                                                                                                                                                                                                                                                                                                                                                                                                      - weka.core.neighboursearch.**TreePerformanceStats**
                                                                                                                                                                                                                                                                                                                                                                                                                                                      - weka.gui.treevisualizer.**PlaceNode1** (implements weka.gui.treevisualizer.NodePlace)- weka.gui.treevisualizer.**PlaceNode2** (implements weka.gui.treevisualizer.NodePlace)- weka.gui.visualize.**PlotData2D**- weka.core.pmml.**PMMLFactory**- weka.core.pmml.**PMMLUtils**- weka.associations.tertius.**Predicate** (implements weka.core.RevisionHandler, java.io.Serializable)- weka.classifiers.trees.adtree.**PredictionNode** (implements java.lang.Cloneable, weka.core.RevisionHandler, java.io.Serializable)- weka.gui.visualize.**PrintableComponent** (implements weka.gui.visualize.PrintableHandler)- weka.classifiers.bayes.blr.**Prior** (implements weka.core.RevisionHandler, java.io.Serializable)
                                                                                                                                                                                                                                                                                                                                                                                                                                                                        - weka.classifiers.bayes.blr.**GaussianPriorImpl**- weka.classifiers.bayes.blr.**LaplacePriorImpl**
                                                                                                                                                                                                                                                                                                                                                                                                                                                                        - weka.associations.**PriorEstimation** (implements weka.core.RevisionHandler, java.io.Serializable)- weka.clusterers.forOPTICSAndDBScan.Utils.**PriorityQueue** (implements weka.core.RevisionHandler)- weka.clusterers.forOPTICSAndDBScan.Utils.**PriorityQueueElement** (implements weka.core.RevisionHandler)- java.beans.PropertyEditorSupport (implements java.beans.PropertyEditor)
                                                                                                                                                                                                                                                                                                                                                                                                                                                                                - weka.gui.**FileEditor**- weka.gui.**SelectedTagEditor**
                                                                                                                                                                                                                                                                                                                                                                                                                                                                                - weka.core.xml.**PropertyHandler** (implements weka.core.RevisionHandler)- weka.experiment.**PropertyNode** (implements weka.core.RevisionHandler, java.io.Serializable)- weka.core.**PropertyPath** (implements weka.core.RevisionHandler)- weka.core.**PropertyPath.Path** (implements weka.core.RevisionHandler)- weka.core.**PropertyPath.PathElement** (implements java.lang.Cloneable, weka.core.RevisionHandler)- weka.core.matrix.**QRDecomposition** (implements weka.core.RevisionHandler, java.io.Serializable)- weka.core.**Queue** (implements weka.core.RevisionHandler, java.io.Serializable)- java.util.Random (implements java.io.Serializable)
                                                                                                                                                                                                                                                                                                                                                                                                                                                                                                - weka.core.**Debug.Random** (implements weka.core.RevisionHandler, java.io.Serializable)- weka.core.**RandomVariates** (implements weka.core.RevisionHandler)
                                                                                                                                                                                                                                                                                                                                                                                                                                                                                                - weka.experiment.**RandomSplitResultProducer** (implements weka.core.AdditionalMeasureProducer, weka.core.OptionHandler, weka.experiment.ResultProducer, weka.core.RevisionHandler)- weka.core.**Range** (implements weka.core.RevisionHandler, java.io.Serializable)- weka.classifiers.functions.supportVector.**RegOptimizer** (implements weka.core.OptionHandler, weka.core.RevisionHandler, java.io.Serializable)
                                                                                                                                                                                                                                                                                                                                                                                                                                                                                                      - weka.classifiers.functions.supportVector.**RegSMO** (implements weka.core.TechnicalInformationHandler)
                                                                                                                                                                                                                                                                                                                                                                                                                                                                                                        - weka.classifiers.functions.supportVector.**RegSMOImproved** (implements weka.core.TechnicalInformationHandler)
                                                                                                                                                                                                                                                                                                                                                                                                                                                                                                      - weka.experiment.**RegressionSplitEvaluator** (implements weka.core.AdditionalMeasureProducer, weka.core.OptionHandler, weka.core.RevisionHandler, weka.experiment.SplitEvaluator)- weka.gui.boundaryvisualizer.**RemoteBoundaryVisualizerSubTask** (implements weka.experiment.Task)- weka.experiment.**RemoteExperimentEvent** (implements java.io.Serializable)- weka.experiment.**RemoteExperimentSubTask** (implements weka.core.RevisionHandler, weka.experiment.Task)- java.rmi.server.RemoteObject (implements java.rmi.Remote, java.io.Serializable)
                                                                                                                                                                                                                                                                                                                                                                                                                                                                                                                - java.rmi.server.RemoteServer
                                                                                                                                                                                                                                                                                                                                                                                                                                                                                                                  - java.rmi.server.UnicastRemoteObject
                                                                                                                                                                                                                                                                                                                                                                                                                                                                                                                    - weka.experiment.**RemoteEngine** (implements weka.experiment.Compute, weka.core.RevisionHandler)
                                                                                                                                                                                                                                                                                                                                                                                                                                                                                                                - weka.gui.boundaryvisualizer.**RemoteResult** (implements java.io.Serializable)- weka.experiment.**ResultMatrix** (implements weka.core.RevisionHandler, java.io.Serializable)
                                                                                                                                                                                                                                                                                                                                                                                                                                                                                                                    - weka.experiment.**ResultMatrixCSV**- weka.experiment.**ResultMatrixGnuPlot**- weka.experiment.**ResultMatrixHTML**- weka.experiment.**ResultMatrixLatex**- weka.experiment.**ResultMatrixPlainText**- weka.experiment.**ResultMatrixSignificance**
                                                                                                                                                                                                                                                                                                                                                                                                                                                                                                                    - weka.gui.sql.**ResultSetHelper**- weka.gui.sql.**ResultSetTableModel** (implements javax.swing.table.TableModel)- weka.core.**RevisionUtils**- weka.associations.tertius.**Rule** (implements java.lang.Cloneable, weka.core.RevisionHandler, java.io.Serializable)- weka.classifiers.rules.**Rule** (implements weka.core.Copyable, weka.core.RevisionHandler, java.io.Serializable, weka.core.WeightedInstancesHandler)- weka.classifiers.trees.m5.**Rule** (implements weka.core.RevisionHandler, java.io.Serializable)- weka.associations.**RuleGeneration** (implements weka.core.RevisionHandler, java.io.Serializable)
                                                                                                                                                                                                                                                                                                                                                                                                                                                                                                                                  - weka.associations.**CaRuleGeneration** (implements weka.core.RevisionHandler, java.io.Serializable)
                                                                                                                                                                                                                                                                                                                                                                                                                                                                                                                                  - weka.associations.**RuleItem** (implements java.lang.Comparable<T>, weka.core.RevisionHandler, java.io.Serializable)- weka.classifiers.rules.**RuleStats** (implements weka.core.RevisionHandler, java.io.Serializable)- weka.gui.**SaveBuffer**- weka.core.mathematicalexpression.**Scanner** (implements java\_cup.runtime.Scanner)- weka.filters.unsupervised.instance.subsetbyexpression.**Scanner** (implements java\_cup.runtime.Scanner)- weka.attributeSelection.**ScatterSearchV1.Subset** (implements java.io.Serializable)- weka.classifiers.bayes.net.search.**SearchAlgorithm** (implements weka.core.OptionHandler, weka.core.RevisionHandler, java.io.Serializable)
                                                                                                                                                                                                                                                                                                                                                                                                                                                                                                                                                - weka.classifiers.bayes.net.search.fixed.**FromFile**- weka.classifiers.bayes.net.search.global.**GlobalScoreSearchAlgorithm**
                                                                                                                                                                                                                                                                                                                                                                                                                                                                                                                                                    - weka.classifiers.bayes.net.search.global.**GeneticSearch**- weka.classifiers.bayes.net.search.global.**HillClimber**
                                                                                                                                                                                                                                                                                                                                                                                                                                                                                                                                                        - weka.classifiers.bayes.net.search.global.**RepeatedHillClimber**- weka.classifiers.bayes.net.search.global.**TabuSearch** (implements weka.core.TechnicalInformationHandler)
                                                                                                                                                                                                                                                                                                                                                                                                                                                                                                                                                        - weka.classifiers.bayes.net.search.global.**K2** (implements weka.core.TechnicalInformationHandler)- weka.classifiers.bayes.net.search.global.**SimulatedAnnealing** (implements weka.core.TechnicalInformationHandler)- weka.classifiers.bayes.net.search.global.**TAN** (implements weka.core.TechnicalInformationHandler)
                                                                                                                                                                                                                                                                                                                                                                                                                                                                                                                                                    - weka.classifiers.bayes.net.search.local.**LocalScoreSearchAlgorithm**
                                                                                                                                                                                                                                                                                                                                                                                                                                                                                                                                                      - weka.classifiers.bayes.net.search.ci.**CISearchAlgorithm**
                                                                                                                                                                                                                                                                                                                                                                                                                                                                                                                                                        - weka.classifiers.bayes.net.search.ci.**ICSSearchAlgorithm**
                                                                                                                                                                                                                                                                                                                                                                                                                                                                                                                                                        - weka.classifiers.bayes.net.search.local.**GeneticSearch**- weka.classifiers.bayes.net.search.local.**HillClimber**
                                                                                                                                                                                                                                                                                                                                                                                                                                                                                                                                                            - weka.classifiers.bayes.net.search.local.**LAGDHillClimber**- weka.classifiers.bayes.net.search.local.**RepeatedHillClimber**- weka.classifiers.bayes.net.search.local.**TabuSearch** (implements weka.core.TechnicalInformationHandler)
                                                                                                                                                                                                                                                                                                                                                                                                                                                                                                                                                            - weka.classifiers.bayes.net.search.local.**K2** (implements weka.core.TechnicalInformationHandler)- weka.classifiers.bayes.net.search.local.**SimulatedAnnealing** (implements weka.core.TechnicalInformationHandler)- weka.classifiers.bayes.net.search.local.**TAN** (implements weka.core.TechnicalInformationHandler)
                                                                                                                                                                                                                                                                                                                                                                                                                                                                                                                                                      - weka.classifiers.bayes.net.search.fixed.**NaiveBayes**
                                                                                                                                                                                                                                                                                                                                                                                                                                                                                                                                                - weka.core.**SelectedTag** (implements weka.core.RevisionHandler)- weka.associations.gsp.**Sequence** (implements java.lang.Cloneable, weka.core.RevisionHandler, java.io.Serializable)- weka.clusterers.forOPTICSAndDBScan.Databases.**SequentialDatabase** (implements weka.clusterers.forOPTICSAndDBScan.Databases.Database, weka.core.RevisionHandler, java.io.Serializable)- weka.core.**SerializationHelper** (implements weka.core.RevisionHandler)- weka.core.**SerializedObject** (implements weka.core.RevisionHandler, java.io.Serializable)- weka.core.xml.**SerialUIDChanger** (implements weka.core.RevisionHandler)- weka.clusterers.forOPTICSAndDBScan.OPTICS\_GUI.**SERObject** (implements weka.core.RevisionHandler, java.io.Serializable)- weka.classifiers.functions.neural.**SigmoidUnit** (implements weka.classifiers.functions.neural.NeuralMethod, weka.core.RevisionHandler)- java.beans.SimpleBeanInfo (implements java.beans.BeanInfo)
                                                                                                                                                                                                                                                                                                                                                                                                                                                                                                                                                                  - weka.gui.beans.**AbstractDataSinkBeanInfo**
                                                                                                                                                                                                                                                                                                                                                                                                                                                                                                                                                                    - weka.gui.beans.**SaverBeanInfo**
                                                                                                                                                                                                                                                                                                                                                                                                                                                                                                                                                                    - weka.gui.beans.**AbstractDataSourceBeanInfo**
                                                                                                                                                                                                                                                                                                                                                                                                                                                                                                                                                                      - weka.gui.beans.**LoaderBeanInfo**
                                                                                                                                                                                                                                                                                                                                                                                                                                                                                                                                                                      - weka.gui.beans.**AbstractTestSetProducerBeanInfo**
                                                                                                                                                                                                                                                                                                                                                                                                                                                                                                                                                                        - weka.gui.beans.**TestSetMakerBeanInfo**
                                                                                                                                                                                                                                                                                                                                                                                                                                                                                                                                                                        - weka.gui.beans.**AbstractTrainAndTestSetProducerBeanInfo**
                                                                                                                                                                                                                                                                                                                                                                                                                                                                                                                                                                          - weka.gui.beans.**CrossValidationFoldMakerBeanInfo**- weka.gui.beans.**TrainTestSplitMakerBeanInfo**
                                                                                                                                                                                                                                                                                                                                                                                                                                                                                                                                                                          - weka.gui.beans.**AbstractTrainingSetProducerBeanInfo**
                                                                                                                                                                                                                                                                                                                                                                                                                                                                                                                                                                            - weka.gui.beans.**TrainingSetMakerBeanInfo**
                                                                                                                                                                                                                                                                                                                                                                                                                                                                                                                                                                            - weka.gui.beans.**AssociatorBeanInfo**- weka.gui.beans.**AttributeSummarizerBeanInfo**- weka.gui.beans.**ClassAssignerBeanInfo**- weka.gui.beans.**ClassifierBeanInfo**- weka.gui.beans.**ClassifierPerformanceEvaluatorBeanInfo**- weka.gui.beans.**ClassValuePickerBeanInfo**- weka.gui.beans.**ClustererBeanInfo**- weka.gui.beans.**ClustererPerformanceEvaluatorBeanInfo**- weka.gui.beans.**CostBenefitAnalysisBeanInfo**- weka.gui.beans.**DataVisualizerBeanInfo**- weka.gui.beans.**FilterBeanInfo**- weka.gui.beans.**GraphViewerBeanInfo**- weka.gui.beans.**IncrementalClassifierEvaluatorBeanInfo**- weka.gui.beans.**InstanceStreamToBatchMakerBeanInfo**- weka.gui.beans.**ModelPerformanceChartBeanInfo**- weka.gui.beans.**PredictionAppenderBeanInfo**- weka.gui.beans.**ScatterPlotMatrixBeanInfo**- weka.gui.beans.**SerializedModelSaverBeanInfo**- weka.gui.beans.**StripChartBeanInfo**- weka.gui.beans.**TextViewerBeanInfo**
                                                                                                                                                                                                                                                                                                                                                                                                                                                                                                                                                                  - weka.gui.**SimpleCLIPanel.CommandlineCompletion**- weka.gui.**SimpleDateFormatEditor** (implements java.beans.PropertyEditor)- weka.associations.tertius.**SimpleLinkedList** (implements weka.core.RevisionHandler, java.io.Serializable)- weka.associations.tertius.**SimpleLinkedList.LinkedListInverseIterator** (implements weka.core.RevisionHandler, java.io.Serializable)- weka.associations.tertius.**SimpleLinkedList.LinkedListIterator** (implements weka.core.RevisionHandler, java.io.Serializable)- weka.core.**SingleIndex** (implements weka.core.RevisionHandler, java.io.Serializable)- weka.core.matrix.**SingularValueDecomposition** (implements weka.core.RevisionHandler, java.io.Serializable)- weka.classifiers.functions.**SMO.BinarySMO** (implements java.io.Serializable)- weka.classifiers.functions.supportVector.**SMOset** (implements weka.core.RevisionHandler, java.io.Serializable)- weka.core.stemmers.**SnowballStemmer** (implements weka.core.OptionHandler, weka.core.stemmers.Stemmer)- weka.gui.**SortedTableModel.SortContainer** (implements java.lang.Comparable<T>)- weka.core.**SpecialFunctions** (implements weka.core.RevisionHandler)- weka.classifiers.trees.j48.**SplitCriterion** (implements weka.core.RevisionHandler, java.io.Serializable)
                                                                                                                                                                                                                                                                                                                                                                                                                                                                                                                                                                                            - weka.classifiers.trees.j48.**EntropyBasedSplitCrit**
                                                                                                                                                                                                                                                                                                                                                                                                                                                                                                                                                                                              - weka.classifiers.trees.j48.**EntropySplitCrit**- weka.classifiers.trees.j48.**GainRatioSplitCrit**- weka.classifiers.trees.j48.**InfoGainSplitCrit**
                                                                                                                                                                                                                                                                                                                                                                                                                                                                                                                                                                                            - weka.classifiers.trees.adtree.**Splitter** (implements java.lang.Cloneable, weka.core.RevisionHandler, java.io.Serializable)
                                                                                                                                                                                                                                                                                                                                                                                                                                                                                                                                                                                              - weka.classifiers.trees.adtree.**TwoWayNominalSplit**- weka.classifiers.trees.adtree.**TwoWayNumericSplit**
                                                                                                                                                                                                                                                                                                                                                                                                                                                                                                                                                                                              - weka.core.neighboursearch.covertrees.**Stack**<T> (implements weka.core.RevisionHandler, java.io.Serializable)- weka.core.**Statistics** (implements weka.core.RevisionHandler)- weka.classifiers.trees.j48.**Stats** (implements weka.core.RevisionHandler)- weka.experiment.**Stats** (implements weka.core.RevisionHandler, java.io.Serializable)- weka.core.stemmers.**Stemming** (implements weka.core.RevisionHandler)- weka.core.**Stopwords** (implements weka.core.RevisionHandler)- weka.gui.**SysErrLog** (implements weka.gui.Logger)- weka.core.**SystemInfo** (implements weka.core.RevisionHandler)- weka.core.**Tag** (implements weka.core.RevisionHandler, java.io.Serializable)- weka.experiment.**TaskStatusInfo** (implements weka.core.RevisionHandler, java.io.Serializable)- weka.core.**TechnicalInformation** (implements weka.core.RevisionHandler)- weka.datagenerators.**Test** (implements weka.core.RevisionHandler, java.io.Serializable)- weka.core.**TestInstances** (implements java.lang.Cloneable, weka.core.OptionHandler, weka.core.RevisionHandler, java.io.Serializable)- weka.classifiers.evaluation.**ThresholdCurve** (implements weka.core.RevisionHandler)- java.lang.Throwable (implements java.io.Serializable)
                                                                                                                                                                                                                                                                                                                                                                                                                                                                                                                                                                                                                            - java.lang.Exception
                                                                                                                                                                                                                                                                                                                                                                                                                                                                                                                                                                                                                              - weka.gui.graphvisualizer.**BIFFormatException**- java.lang.RuntimeException
                                                                                                                                                                                                                                                                                                                                                                                                                                                                                                                                                                                                                                  - weka.core.**UnassignedClassException**- weka.core.**UnassignedDatasetException**
                                                                                                                                                                                                                                                                                                                                                                                                                                                                                                                                                                                                                                  - weka.core.**WekaException**
                                                                                                                                                                                                                                                                                                                                                                                                                                                                                                                                                                                                                                    - weka.core.**NoSupportForMissingValuesException**- weka.core.**UnsupportedAttributeTypeException**- weka.core.**UnsupportedClassTypeException**
                                                                                                                                                                                                                                                                                                                                                                                                                                                                                                                                                                                                                            - weka.core.tokenizers.**Tokenizer** (implements java.util.Enumeration<E>, weka.core.OptionHandler, weka.core.RevisionHandler, java.io.Serializable)
                                                                                                                                                                                                                                                                                                                                                                                                                                                                                                                                                                                                                              - weka.core.tokenizers.**AlphabeticTokenizer**- weka.core.tokenizers.**CharacterDelimitedTokenizer**
                                                                                                                                                                                                                                                                                                                                                                                                                                                                                                                                                                                                                                  - weka.core.tokenizers.**NGramTokenizer**- weka.core.tokenizers.**WordTokenizer**
                                                                                                                                                                                                                                                                                                                                                                                                                                                                                                                                                                                                                              - weka.gui.treevisualizer.**TreeBuild**- weka.gui.treevisualizer.**TreeDisplayEvent**- weka.core.**Trie** (implements java.lang.Cloneable, java.util.Collection<E>, weka.core.RevisionHandler, java.io.Serializable)- weka.core.**Trie.TrieIterator** (implements java.util.Iterator<E>, weka.core.RevisionHandler)- weka.classifiers.evaluation.**TwoClassStats** (implements weka.core.RevisionHandler)- weka.clusterers.forOPTICSAndDBScan.Utils.**UpdateQueue** (implements weka.core.RevisionHandler)- weka.clusterers.forOPTICSAndDBScan.Utils.**UpdateQueueElement** (implements weka.core.RevisionHandler)- weka.core.**Utils** (implements weka.core.RevisionHandler)- weka.classifiers.trees.m5.**Values** (implements weka.core.RevisionHandler)- weka.classifiers.bayes.net.**VaryNode** (implements weka.core.RevisionHandler, java.io.Serializable)- weka.core.**Version** (implements java.lang.Comparable<T>, weka.core.RevisionHandler)- weka.gui.visualize.**VisualizePanelEvent**- weka.gui.visualize.**VisualizeUtils**- weka.core.xml.**XMLDocument** (implements weka.core.RevisionHandler)
                                                                                                                                                                                                                                                                                                                                                                                                                                                                                                                                                                                                                                                          - weka.core.xml.**XMLInstances** (implements java.io.Serializable)
                                                                                                                                                                                                                                                                                                                                                                                                                                                                                                                                                                                                                                                          - weka.core.xml.**XMLOptions** (implements weka.core.RevisionHandler)- weka.core.xml.**XMLSerialization** (implements weka.core.RevisionHandler)
                                                                                                                                                                                                                                                                                                                                                                                                                                                                                                                                                                                                                                                              - weka.core.xml.**XMLBasicSerialization**
                                                                                                                                                                                                                                                                                                                                                                                                                                                                                                                                                                                                                                                                - weka.gui.beans.xml.**XMLBeans**- weka.classifiers.xml.**XMLClassifier**- weka.experiment.xml.**XMLExperiment**
                                                                                                                                                                                                                                                                                                                                                                                                                                                                                                                                                                                                                                                              - weka.core.xml.**XMLSerializationMethodHandler** (implements weka.core.RevisionHandler)- weka.core.xml.**XStream** (implements weka.core.RevisionHandler)- weka.classifiers.trees.m5.**YongSplitInfo** (implements java.lang.Cloneable, weka.core.RevisionHandler, java.io.Serializable, weka.classifiers.trees.m5.SplitEvaluate)

## Interface Hierarchy

- weka.core.**AdditionalMeasureProducer**- weka.associations.**Associator**- weka.attributeSelection.**AttributeEvaluator**- weka.gui.visualize.**AttributePanelListener**- weka.attributeSelection.**AttributeTransformer**- weka.core.converters.**BatchConverter**- weka.gui.beans.**BeanCommon**- weka.core.**CapabilitiesHandler**
                - weka.core.**MultiInstanceCapabilitiesHandler**
                - weka.clusterers.**Clusterer**
                  - weka.clusterers.**DensityBasedClusterer**
                  - weka.gui.beans.**ConnectionNotificationConsumer**- weka.core.**Copyable**- weka.gui.beans.**CustomizerCloseRequester**- weka.gui.beans.**CustomizerClosingListener**- weka.gui.**CustomPanelSupplier**- weka.clusterers.forOPTICSAndDBScan.Databases.**Database**- weka.core.converters.**DatabaseConverter**- weka.gui.beans.**DataFormatListener**- weka.gui.boundaryvisualizer.**DataGenerator**- weka.clusterers.forOPTICSAndDBScan.DataObjects.**DataObject**- weka.gui.beans.**DataSink**- weka.gui.beans.**DataSource**- weka.core.**Drawable**- weka.core.**EnvironmentHandler**- weka.attributeSelection.**ErrorBasedMeritEvaluator**- weka.gui.beans.**EventConstraints**- java.util.EventListener
                                                    - weka.gui.beans.**BatchClassifierListener**- weka.gui.beans.**BatchClustererListener**- weka.gui.beans.**ChartListener**- weka.gui.sql.event.**ConnectionListener**- weka.gui.beans.**DataSourceListener**- weka.gui.explorer.**Explorer.CapabilitiesFilterChangeListener**- weka.gui.beans.**GraphListener**- weka.gui.sql.event.**HistoryChangedListener**- weka.gui.beans.**IncrementalClassifierListener**- weka.gui.beans.**InstanceListener**- weka.gui.streams.**InstanceListener**- weka.gui.sql.event.**QueryExecuteListener**- weka.gui.sql.event.**ResultChangedListener**- weka.gui.streams.**SerialInstanceListener**- weka.gui.beans.**StartUpListener**- weka.gui.beans.**TestSetListener**- weka.gui.beans.**TextListener**- weka.gui.beans.**ThresholdDataListener**- weka.gui.beans.**TrainingSetListener**- weka.gui.beans.**VisualizableErrorListener**
                                                    - weka.gui.explorer.**Explorer.ExplorerPanel**- weka.gui.explorer.**Explorer.LogHandler**- weka.core.converters.**FileSourcedConverter**- weka.gui.graphvisualizer.**GraphConstants**- weka.core.converters.**IncrementalConverter**- weka.estimators.**IncrementalEstimator**- weka.gui.streams.**InstanceProducer**- weka.classifiers.**IntervalEstimator**- weka.classifiers.**IterativeClassifier**- weka.core.**JythonObject**
                                                                        - weka.core.**JythonSerializableObject** (also extends java.io.Serializable)
                                                                        - weka.classifiers.lazy.kstar.**KStarConstants**- weka.gui.graphvisualizer.**LayoutCompleteEventListener**- weka.gui.graphvisualizer.**LayoutEngine**- weka.gui.**Logger**- weka.gui.beans.**LogWriter**- weka.gui.**MainMenuExtension**- weka.core.**Matchable**- weka.gui.treevisualizer.**NodePlace**- weka.clusterers.**NumberOfClustersRequestable**- weka.core.**OptionHandler**
                                                                                            - weka.associations.**CARuleMiner**- weka.core.**DistanceFunction**
                                                                                            - weka.gui.visualize.**Plot2DCompanion**- weka.core.pmml.**PMMLModel**- weka.classifiers.evaluation.**Prediction**- weka.gui.visualize.**PrintableHandler**- weka.core.**Randomizable**- weka.attributeSelection.**RankedOutputSearch**- java.rmi.Remote
                                                                                                          - weka.experiment.**Compute**
                                                                                                          - weka.experiment.**RemoteExperimentListener**- weka.core.**RevisionHandler**
                                                                                                              - weka.estimators.**ConditionalEstimator**- weka.core.converters.**Loader** (also extends java.io.Serializable)- weka.core.converters.**Saver** (also extends java.io.Serializable)- weka.core.stemmers.**Stemmer** (also extends java.io.Serializable)
                                                                                                              - weka.classifiers.bayes.net.search.local.**Scoreable**- java.io.Serializable
                                                                                                                  - weka.core.**JythonSerializableObject** (also extends weka.core.JythonObject)- weka.core.converters.**Loader** (also extends weka.core.RevisionHandler)- weka.classifiers.functions.neural.**NeuralMethod**- weka.experiment.**ResultListener**- weka.experiment.**ResultProducer**- weka.core.converters.**Saver** (also extends weka.core.RevisionHandler)- weka.experiment.**SplitEvaluator**- weka.core.stemmers.**Stemmer** (also extends weka.core.RevisionHandler)- weka.experiment.**Task**- weka.experiment.**Tester**
                                                                                                                  - weka.classifiers.**Sourcable**- weka.filters.**Sourcable**- weka.classifiers.trees.m5.**SplitEvaluate**- weka.gui.beans.**Startable**- weka.attributeSelection.**StartSetHandler**- weka.filters.**StreamableFilter**- weka.gui.beans.**StructureProducer**- weka.attributeSelection.**SubsetEvaluator**- weka.core.**Summarizable**- weka.filters.**SupervisedFilter**- weka.core.mathematicalexpression.**sym**- weka.filters.unsupervised.instance.subsetbyexpression.**sym**- weka.gui.**TaskLogger**- weka.core.**TechnicalInformationHandler**- weka.gui.beans.**TestSetProducer**- weka.gui.beans.**TrainingSetProducer**- weka.gui.treevisualizer.**TreeDisplayListener**- weka.core.**Undoable**- weka.filters.**UnsupervisedFilter**- weka.classifiers.**UpdateableClassifier**- weka.clusterers.**UpdateableClusterer**- weka.core.converters.**URLSourcedLoader**- weka.gui.beans.**UserRequestAcceptor**- weka.gui.beans.**Visible**- weka.gui.visualize.**VisualizePanelListener**- weka.gui.visualize.plugins.**VisualizePlugin**- weka.core.**WeightedInstancesHandler**- weka.gui.beans.**WekaWrapper**

## Enum Hierarchy

- java.lang.Object
  - java.lang.Enum<E> (implements java.lang.Comparable<T>, java.io.Serializable)
    - weka.associations.**FPGrowth.AssociationRule.METRIC\_TYPE**- weka.core.**TechnicalInformation.Type**- weka.core.**TechnicalInformation.Field**- weka.core.**RevisionUtils.Type**- weka.core.**Capabilities.Capability**- weka.core.logging.**Logger.Level**- weka.core.pmml.**FieldMetaInfo.Value.Property**- weka.core.pmml.**FieldMetaInfo.Interval.Closure**- weka.core.pmml.**FieldMetaInfo.Optype**

---


|  |  |  |
| --- | --- | --- |
| **Overview** \| Package \| Class \| **Tree** \| **Deprecated** \| **Index** \| **Help** \| **Weka's home** | |  |
| PREV   NEXT | **FRAMES**    **NO FRAMES**     **All Classes** |  |


---
